# A Semi-automated Organoid Screening Method Demonstrates Epigenetic Control of Intestinal Epithelial Differentiation

**DOI:** 10.3389/fcell.2020.618552

**Published:** 2021-01-21

**Authors:** Jenny Ostrop, Rosalie T. Zwiggelaar, Marianne Terndrup Pedersen, François Gerbe, Korbinian Bösl, Håvard T. Lindholm, Alberto Díez-Sánchez, Naveen Parmar, Silke Radetzki, Jens Peter von Kries, Philippe Jay, Kim B. Jensen, Cheryl Arrowsmith, Menno J. Oudhoff

**Affiliations:** ^1^Centre of Molecular Inflammation Research (CEMIR), Department of Clinical and Molecular Medicine (IKOM), NTNU - Norwegian University of Science and Technology, Trondheim, Norway; ^2^BRIC - Biotech Research and Innovation Centre, University of Copenhagen, Copenhagen, Denmark; ^3^Novo Nordisk Foundation Center for Stem Cell Biology, Faculty of Health and Medical Sciences, University of Copenhagen, Copenhagen, Denmark; ^4^Cancer Biology Department, Institute of Functional Genomics, University of Montpellier, Montpellier, France; ^5^Department of Bioinformatics, Computational Biological Unit, University of Bergen, Bergen, Norway; ^6^Screening Unit, Leibniz-Forschungsinstitut für Molekulare Pharmakologie, Berlin, Germany; ^7^Structural Genomics Consortium, University of Toronto, Toronto, ON, Canada; ^8^Princess Margaret Cancer Centre, University Health Network, Toronto, ON, Canada; ^9^Department of Medical Biophysics, University of Toronto, Toronto, ON, Canada

**Keywords:** organoids, epigenetic modifiers, intestinal stem cell biology, bioimage analysis, PRMT1, EP300, CREBBP

## Abstract

Intestinal organoids are an excellent model to study epithelial biology. Yet, the selection of analytical tools to accurately quantify heterogeneous organoid cultures remains limited. Here, we developed a semi-automated organoid screening method, which we applied to a library of highly specific chemical probes to identify epigenetic regulators of intestinal epithelial biology. The role of epigenetic modifiers in adult stem cell systems, such as the intestinal epithelium, is still undefined. Based on this resource dataset, we identified several targets that affected epithelial cell differentiation, including HDACs, EP300/CREBBP, LSD1, and type I PRMTs, which were verified by complementary methods. For example, we show that inhibiting type I PRMTs, which leads enhanced epithelial differentiation, blocks the growth of adenoma but not normal organoid cultures. Thus, epigenetic probes are powerful tools to study intestinal epithelial biology and may have therapeutic potential.

## Introduction

The intestinal epithelium, a single layer of cells, faces the challenge of both providing a barrier against pathogens while also being responsible for the uptake of nutrients and water. One of the hallmarks of intestinal epithelium is the rapid turnover of 3–5 days, which is driven by LGR5+ intestinal stem cells (ISCs) that reside at the bottom of crypts. ISCs are continuously dividing and give rise to progenitor cells, which differentiate into specialized intestinal epithelial cell (IEC) lineages such as absorptive enterocytes and secretory lineages such as mucus-producing goblet cells, antimicrobial-producing Paneth cells, hormone-secreting enteroendocrine cells, and chemosensory tuft cells (Gehart and Clevers, 2019). The intestinal epithelium exhibits high plasticity in respond to challenges (Haber et al., 2017; Jadhav et al., 2017). On the other hand, it is vulnerable to tumorigenesis with colorectal cancer being the second leading cause of cancer-related deaths worldwide.

The balance between ISC proliferation and IEC differentiation is controlled by pathways including WNT, BMP, and NOTCH (Gehart and Clevers, 2019). Specific transcription factors, such as ATOH1, are critically required for acquisition of IEC effector lineage identities (Yang et al., 2001). Gene expression is further determined by the chromatin landscape. It is known that epigenetic marks such as methylated DNA and histone tail modifications differ strongly between fetal and adult intestine (Kazakevych et al., 2017; Jadhav et al., 2019), and can be altered in intestinal pathologies (Ray and Longworth, 2019). While the requirement of epigenetic modifications for embryonic stem cell differentiation (Atlasi and Stunnenberg, 2017) and differentiation and maturation of immune cells (Álvarez-Errico et al., 2015) has been extensively studied, their role for maintenance of intestinal homeostasis is debated. Both a permissive chromatin structure and regulation of IEC lineage differentiation by transcription factors, and a control of gene expression patterns by the chromatin states itself have been proposed as conflicting models (extensively reviewed by Elliott and Kaestner, 2015). The classic NOTCH-mediated lateral inhibition model of ISC-to-IEC differentiation has been attributed to a broadly permissive chromatin landscape, supporting the idea of regulation by transcription factors as the most defining factor (Kim et al., 2014). However, other studies suggest that ISC differentiation and the de-differentiation of lineage-defined IECs back to ISCs are mediated by changes in DNA methylation and chromatin accessibility (Kaaij et al., 2013; Sheaffer et al., 2014; Jadhav et al., 2017; Kazakevych et al., 2017). Several hundred epigenetic modification enzymes contribute to writing, erasing, and reading the epigenetic code (Arrowsmith et al., 2012). Currently, the investigation of the role of epigenetic modifiers in the intestinal epithelium depends mostly on labor-intensive mouse models with conditional genetic deletion, allowing for the examination of one or few epigenetic modifiers at the same time (Gonneaud et al., 2016b; Koppens et al., 2016). A higher throughput could be achieved by using organoids to investigate epigenetic effects in the intestinal epithelium (Kraiczy and Zilbauer, 2019). Curated by the Structural Genomics Consortium, an openly accessible chemical probe library targeting epigenetic modification enzymes with high selectivity and specificity became available recently (Ackloo et al., 2017; Scheer et al., 2019). Treating organoids with this chemical probe library will enable a direct comparison of the putative requirement of many epigenetic modifiers for epithelial homeostasis or differentiation of IEC lineages.

Heterogeneous organoid cultures are quite sensitive to subtle changes in handling and culture conditions. Therefore, development of quantitative analysis methods for reproducible quantification of a whole organoid population instead of relying on representative example data points is crucial (Huch et al., 2017). Indeed, this has recently led to specialized studies such as using light-sheet microscopy to elegantly define symmetry breaking (Serra et al., 2019), using single-cell RNA sequencing (scRNA-seq) to describe epithelial responses to immune cues (Haber et al., 2017; Biton et al., 2018), or analysis of single intestinal organoids in microcavity arrays (Brandenberg et al., 2020). However, these techniques are costly and the required instrumentation and data analysis pipelines are not widely available to the research community. Thus, quantitative but cost-efficient tools based on standard laboratory equipment that can be scaled to screen setups need to be established.

Here, we provide a semi-automated organoid quantification method suitable for screening experiments and designed to be used in laboratories with a standard infrastructure. To widely investigate the role of epigenetic modifiers for adult intestinal epithelial homeostasis, we combined this toolbox with a chemical probe library consisting of 39 inhibitors that target epigenetic modification enzymes with high selectivity and specificity (Scheer et al., 2019). From this screen dataset, we identified several mediators of IEC biology that we verified with complementary methods. We envision that this resource will be useful for the research community and will lay basis for further mechanistic investigation. Specifically, we find new regulators of organoid size related to ISC frequency, as well as new regulators of IEC differentiation. Finally, we explore the potential of some of these probes for treatment of intestinal cancer by application on intestinal tumor organoids.

## Results

### Development of a Toolbox to Quantify Intestinal Organoid Growth and Cellular Composition

**Figure 1:**
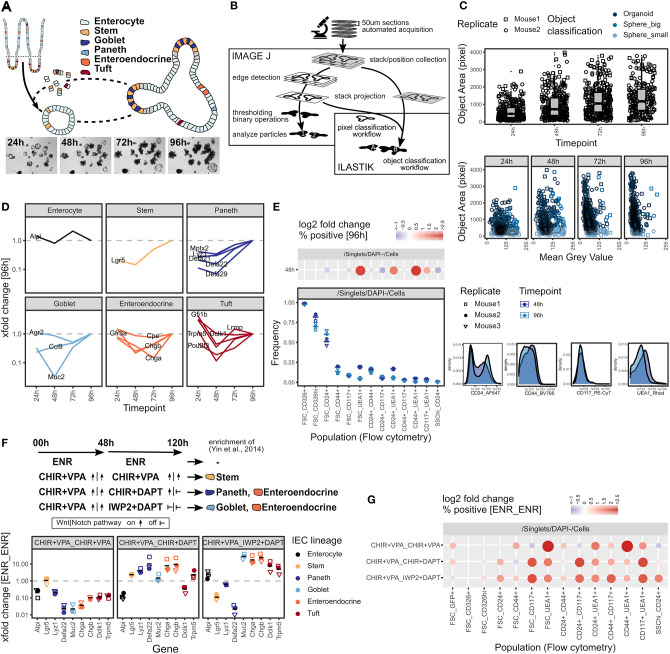
Quantification of intestinal organoid growth and cellular composition. **(A)** Scheme of organoid formation and images of a representative position 24–96 h after seeding. Whole well is shown in Supplementary Figure 1a. **(B)** Scheme of organoid size quantification workflow using open-source tools ImageJ/Fiji and Ilastik. Visual quantification output and ImageJ quantification results are shown in Supplementary Figures 1b,c. **(C)** Box plots showing organoid size quantified as object area at 24–96 h timepoints (top). Object area vs. object mean gray value (8-bit scale) on a minimum projection of the image stack (bottom). Pooled data from 2 biol. replicates, indicated by shape. Each dot represents one organoid. **(D)** mRNA expression of IEC lineage marker genes at 24–96 h timepoints, measured by qRT-PCR. xfold change relative to 96 h organoids, median of 3 biol. replicates. **(E)** Flow cytometry of organoids grown for 48 and 96 h. Staining of representative replicate (bottom right). Population frequencies in Cells parent gate, 3 biol. replicates, indicated by shape. Mean highlighted (bottom left). Log2 fold change relative to 96 h timepoint, median of 3 biol. replicates. Dot size corresponds to absolute log2 fold change (top left). Gating strategy and population frequencies for FSC_CD326^hi^ and FSC_CD24+ parent gates are shown in Supplementary Figures 1d,e. **(F)** Organoids cultured for 48 h followed by 72 h (48 h_72 h) with normal culture medium (ENR_ENR), or culture medium containing CHIR+VPA_CHIR+VPA, CHIR+VPA_CHIR+DAPT, or CHIR+VPA_IWP2+DAPT to modify IEC composition by interfering with Wnt and Notch signaling pathways as indicated, adapted from Yin et al. (2014). mRNA expression measured by qRT-PCR. xfold change relative to ENR_ENR treatment. 3 biol. replicates, indicated by shape. Mean highlighted. **(G)** Flow cytometry of organoids treated for 48_72h as indicated, population frequencies normalized to ENR_ENR treatment. Log2 fold change, median of 3 biol. replicates. Dot size corresponds to absolute log2 fold change. Gating strategy, representative staining and population frequencies for FSC_CD326^hi^ and FSC_CD24+ parent gates are shown in Supplementary Figures 1d,f–h.

A decade after its establishment by Sato et al. (2009), the use of intestinal organoids has been become a standard in the method repertoire. However, accurately quantifying heterogeneous organoid cultures remains a challenge and the analytical tools available to a broad community, especially for screening purposes, remain limited or labor intensive. We thus initiated a small intestinal (SI) organoid system that, similar to the original work, starts with freshly isolated crypts that self-organize into budding organoids by day 4 (96 h after seeding), which can be split and propagated (Figure 1A, Supplementary Figure 1a). We next designed a setup to daily acquire bright-field z-stack images of the whole extracellular matrix (Matrigel droplet) in a well, followed by automatic segmentation and quantification of all individual organoids based on the open-source tools ImageJ/Fiji and Ilastik (Figure 1B). Based on edge detection in each stack layer, this workflow can be used to robustly quantify organoid size (object area) and classify, e.g., by determining intensity, organoid phenotypes over time (Figure 1C, Supplementary Figures 1b,c). The workflow is robust to changes in morphology, stitching artifacts, and can be adjusted to image data from different automated microscopes. As the object classification by Ilastik is not dependent on deep learning and extensive training data, it can easily be adapted to new phenotypes and changes in experimental conditions.

In addition to determining organoid size, the cellular composition is of critical interest. We therefore selected transcripts that are specific for individual IEC lineages (Haber et al., 2017), and performed qRT-PCR on these within a 24–96 h time course (Figure 1D). Except for the enterocyte marker *Alpi*, we generally find an increase in lineage-specific gene expression over time cumulating at 96 h (Figure 1D). This corresponds to the transition from spheroids, consisting mainly of progenitors, to mature budding organoids that contain more differentiated lineages, as was shown previously (Serra et al., 2019). As a complementary technique to quantify cellular composition on a single-cell level, we conducted flow cytometry of commonly used IEC surface markers (Figure 1E, Supplementary Figures 1d,e). The differences between 48 and 96 h organoids were modest (Figure 1E). Of note, we observed that the frequency of *Ulex europaeus* agglutinin 1 (UEA1) positive cells reduced over time, indicating that the population expressing UEA1 on the surface may be progenitor cells that are different from the population of UEA1^bright^ secretory cells commonly detected by immunofluorescence staining of permeabilized tissue (Figure 1E). As a proof of principle, we next tested our approach on organoids with an altered cell composition. Interfering with WNT and NOTCH signaling pathways has previously been established by Yin and colleagues as a method to enrich organoids for stem cells, Paneth cells, goblet cells, or enteroendocrine cells (Yin et al., 2014). WNT and NOTCH pathways are activated or respectively inhibited by treatment with combinations of the glycogen synthase kinase 3 (GSK3) inhibitor CHIR99021 (CHIR), valproic acid (VPA), the porcupine inhibitor IWP2, or the gamma-secretase inhibitor DAPT (Yin et al., 2014) (Figure 1F). Interestingly, we found that incubation with CHIR + VPA followed by IWP2 + DAPT increased the expression of tuft cell marker genes in addition to the previously described effects on goblet cells and enteroendocrine cells (Figure 1F). Drastic effects on the cell composition were reflected by widely altered surface marker expression measured by flow cytometry and resulted in characteristic patterns (Figure 1G, Supplementary Figures 1d,f,g). However, we observed that well established flow cytometry gating strategies, such as identifying Paneth cells by a SSC^hi^_CD24+ gate (Sato et al., 2011), did not follow the *Lyz1* gene expression pattern in some conditions (Figure 1F, Supplementary Figure 1h). Thus, while flow cytometry demonstrates to be very useful to detect changes in the organoid composition, surface marker expression may be influenced by additional factors, such as the growth conditions, and identification of certain cell populations by flow cytometry requires appropriate controls. In summary, we developed an easy-to-use and cost-efficient toolbox for the analysis of (intestinal) organoids that is suitable to detect changes in organoid growth and cell composition.

### Organoid Screen of Epigenetic Modifier Inhibitors Identifies Established Drugs Targeting Cancer Growth

**Figure 2:**
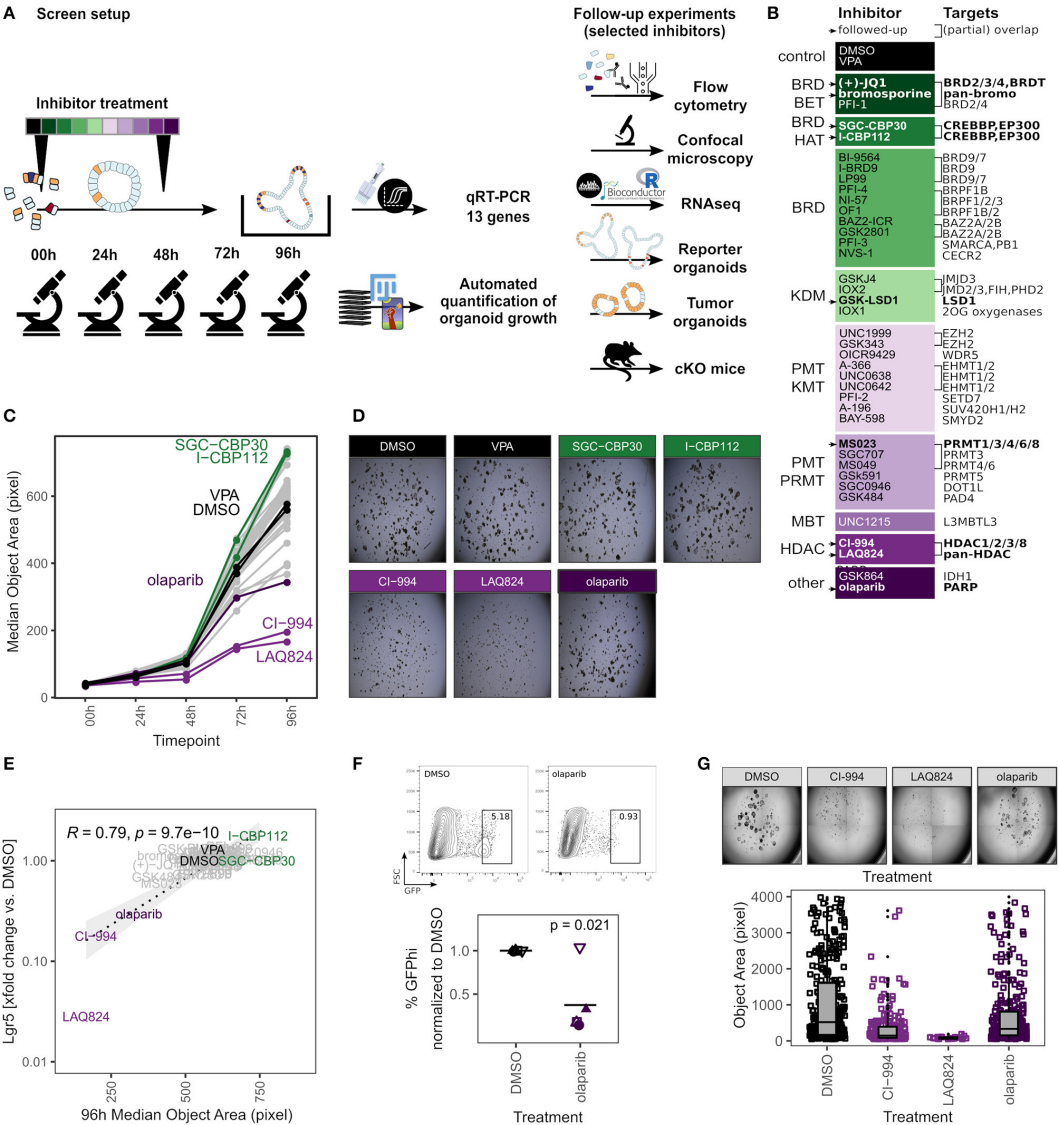
Organoid screen of epigenetics probes identifies established cancer drugs. **(A)** Scheme of screen setup and follow-up experiments. **(B)** Inhibitors used in screen, detailed information is provided in Supplementary Table 1. Probes used in follow-up experiments are highlighted. Abbreviations of inhibitor target families (inhibitor class): BRD, bromodomain; BET, bromodomain and extra-terminal motif; HAT, histone acetyltransferase; KDM, lysine demethylase; PMT, protein methyltransferase; KMT, lysine methyltransferase; PRMT, protein arginine methyltransferase; MBT, malignant brain tumor; HDAC, histone deacetylase. **(C)** Median object area of organoids treated with DMSO or inhibitors for 0–96 h. Median of 4 biol. replicates. Boxplots for each inhibitor and timepoint are shown in Supplementary Figure 2a. Probes that altered organoid size and were followed-up are highlighted: Controls, CI-994, LAQ824 (HDAC inhibitors), olaparib (PARP inhibitor), SGC-CBP30, I-CBP112 (BRD_BET inhibitors). **(D)** Representative replicates, 96 h timepoint. **(E)** Correlation of median organoid size and relative *Lgr5* gene expression, median of 4 biol. replicates. Pearson coefficient. **(F)** Frequency of *Lgr5*-EGFP stem cells in reporter organoids treated with DMSO or olaparib for 96 h. Gating of representative replicate (top) and percentage of GFP^hi^ cells, normalized to DMSO control. 5 biol. replicates, indicated by shape. Mean highlighted. Minimum 5,000 viable cells in parent gate. Paired *t*-test (bottom). Percentage of total GFP+ cells is shown in Supplementary Figure 2d. **(G)** *Apc*-deficient adenomas treated with DMSO, CI-994, LAQ824, or olaparib for 96 h. Representative replicate (top) and quantification of object size in 7/3/3/7 (DMSO/CI-994/LAQ828/olaparib) individual wells (bottom).

Next, we applied our organoid toolbox for screening of a chemical probe library that targets epigenetic modifiers to modulate the epigenome (Scheer et al., 2019). Organoids generated from 4 individual mice were grown in the presence of 39 inhibitors, with DMSO vehicle and VPA serving as controls (Figures 2A,B). Samples were imaged daily and expression of 12 transcripts specific for IEC lineages (Haber et al., 2017) was analyzed at the 96 h endpoint (Figure 2A). We observed that some of the probes significantly affected organoid growth as determined by area (Figures 2C,D, Supplementary Figures 2a–c, 3A). Integration of the primary readouts revealed a strong correlation of organoid size and expression of the ISC marker gene *Lgr5* (Figure 2E). We found three probes that reduced both organoid size and *Lgr5* mRNA expression, namely the pan-Poly (ADP-ribose) polymerase (PARP) inhibitor olaparib and two histone deacetylase (HDAC) inhibitors LAQ824 (Dacinostat), a pan-HDAC inhibitor, and CI-994 (Tacedinaline), an HDAC1-3 and HDAC8 inhibitor (Figures 2C–E, Supplementary Figures 2b,c) (Weisberg et al., 2004; Beckers et al., 2007; Mateo et al., 2019). These findings are in agreement with a study that showed reduced growth and *Lgr5* gene expression but a gain of enterocyte marker expression in CI-994 treated organoids (Gonneaud et al., 2016a). Olaparib-treated organoids would sufficiently grow to perform flow cytometry. This allowed us to use *Lgr5*-EGFP expressing reporter organoids to confirm the reduced *Lgr5* gene expression levels. Indeed, we found markedly fewer GFP-high/GFP-positive cells in olaparib-treated compared to control organoids (Figure 2F, Supplementary Figure 2d). Finally, we treated Adenomatous polyposis coli (*Apc*) knockout organoids, which are a model for intestinal cancer, with the two HDAC inhibitors and olaparib and found that these probes also limited growth in these tumor cultures, with similar growth reductions compared to WT organoids (Figure 2G). Together, this is well in line with the design goal of these probes to limit cellular growth to target cancer cells.

**Figure 3:**
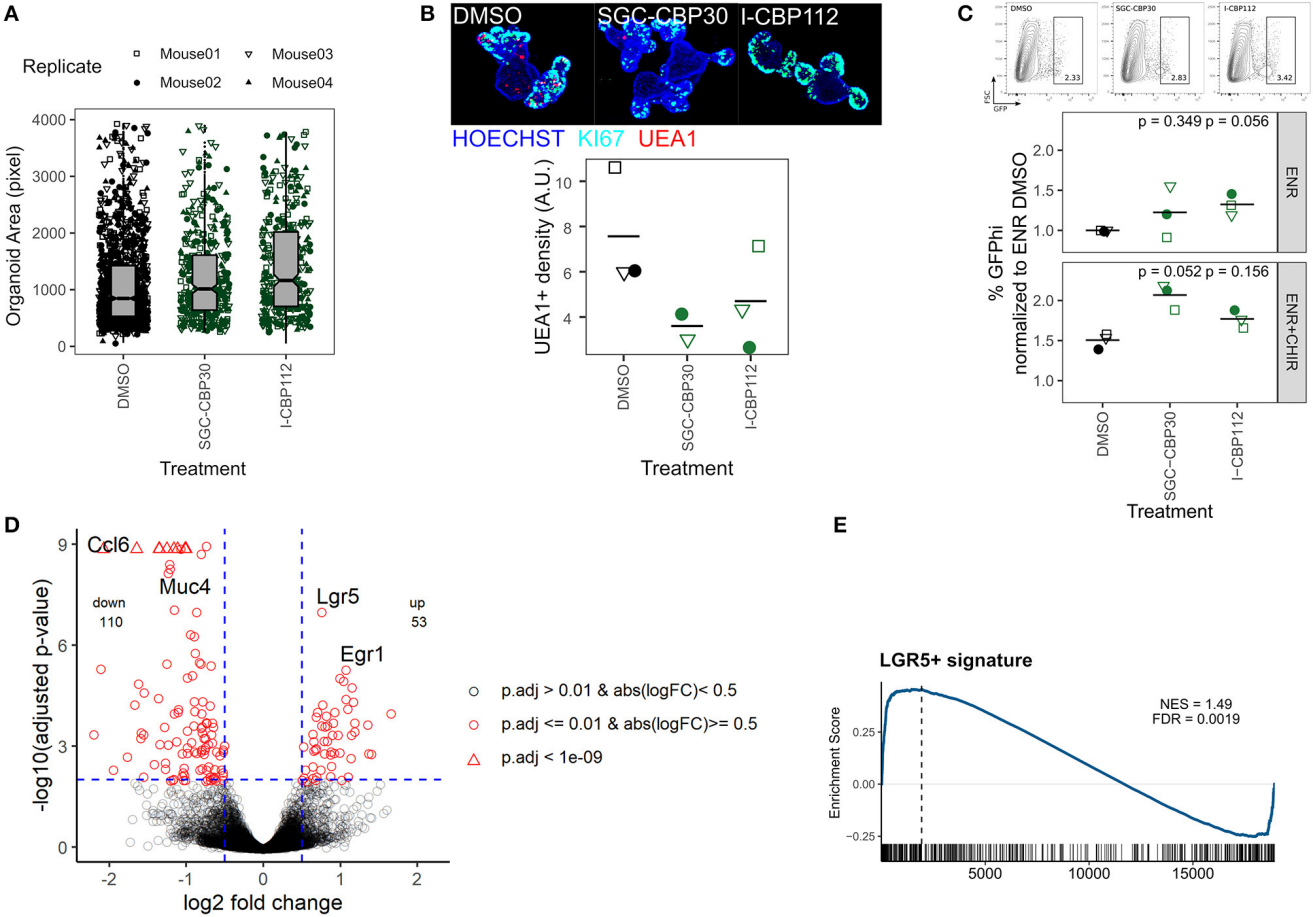
Inhibition of EP300/CREBBP enhances organoid size and *Lgr5* expression. **(A)** Organoids treated with DMSO, SCG-CBP30, or I-CBP112 for 96 h. Area of objects classified as “Organoid” by combined ImageJ/Ilastik workflow. 4 biol. replicates, indicated by shape. **(B)** Representative organoids treated with DMSO, SCG-CBP30, or I-CBP112 for 96 h. 10x magnification, max. intensity projection. KI67 staining marks crypt regions (top). Density of UEA1+ cells, each value represents the median of ≥5 organoids quantified. 3/2/3 biol. replicates, indicated by shape. Mean highlighted (bottom). Full wells for one representative replicate is shown in Supplementary Figure 3c. **(C)** Frequency of *Lgr5*-EGFP stem cells in reporter organoids grown in ENR or ENR+CHIR and treated with DMSO, SCG-CBP30, or I-CBP112 for 96 h, measured by flow cytometry. Gating of representative replicate grown in ENR (top) and percentage of GFP^hi^ cells normalized to ENR DMSO condition of 3 biol. replicates, indicated by shape. Mean highlighted. Paired *t*-test (bottom). Percentage of total GFP+ cells is shown in Supplementary Figure 3d. **(D)** Volcano plot of mRNA sequencing of untreated vs. I-CBP112 treated organoids, 4 biol replicates per group. Selected genes are highlighted. **(E)** mRNA sequencing of untreated vs. I-CBP112 treated organoids. GSEA of LGR5+ stem cell signature from Muñoz et al. (2012) (GSE33949). Normalized enrichment score (NES) and false discovery rate (FDR) are indicated.

### Inhibition of EP300/CREBBP Enhances Organoid Size and *Lgr5* Expression

We next focused on probes that increased organoid size (Figures 2B–D). We found that both SGC-CBP30 and I-CBP112 significantly increased the organoid area (Supplementary Figure 3a) and this increase was seen in objects that were classified as “Organoids” and thus was not dependent on the occurrence of large spheres (Figure 3A, Supplementary Figure 3b). In support of the sensitivity of our assay, both probes have the same targets: EP300/CREBBP. E1A Binding Protein P300 (EP300, P300) and Creb-binding protein (CREBBP, CBP) are closely related bromodomain-containing acetyltransferases that serve as transcriptional co-activators for numerous transcription factors (Goodman and Smolik, 2000; Ramos et al., 2010; Raisner et al., 2018). Both SCG-CBP30 and I-CBP112 specifically target the bromodomain-binding domain, which thus renders these proteins unable to bind acetylated lysines. The observed increase in organoid size was surprising since both inhibitors have been designed to cause growth restriction in cancer cells (Hay et al., 2014; Picaud et al., 2015; Attar and Kurdistani, 2017). Comparing SGC-CBP30/I-CBP112-treated organoids with the DMSO vehicle control, organoid morphology appeared normal, however, we observed a reduction of putative goblet/Paneth cells as determined by cytosolic UEA1 staining (Figure 3B, Supplementary Figure 3c). We next tested whether these probes would expand the LGR5+ cell population in *Lgr5*-EGFP organoids and found a modest increase upon treatment, either alone or in combination with our positive control CHIR, an activator of canonical WNT signaling (Figure 3C, Supplementary Figures 3d,e). However, incubation with SGC-CBP30 or I-CBP112 could not enhance organoid growth under low EGF concentrations, replace R-Spondin in the culture medium, or overcome treatment with the WNT inhibitor IWP2 (Supplementary Figures 3f,g). To determine which genes are under the control of EP300/CREBBP in the intestinal epithelium, we performed mRNA sequencing on untreated vs. I-CBP112 treated organoid cultures. In accordance with a transcriptional co-activator role for EP300/CREBBP, we found 53 genes upregulated and 110 genes downregulated using a log2 fold change cutoff of 0.5 and padjust ≤ 0.01 (Figure 3D). Furthermore, signatures of transcription factors known to interact with either EP300 or CREBBP were negatively enriched (Supplementary Figure 3h). Remarkably, *Lgr5* was the most significantly upregulated gene in our dataset, substantiating our previous results (Figure 3D). In support, gene set enrichment analysis (GSEA) with a LGR5+ stem cell gene set (Muñoz et al., 2012) showed positive correlation (Figure 3E). The second most significantly upregulated gene was *Egr1* (Figure 3D), which is an inducible transcription factor that is involved in cell proliferation (Gitenay and Baron, 2009). The expansion of ISCs or progenitors appears to come at a cost to the differentiation of other cell lineages. We observed reduced UEA1 staining and downregulation of goblet cell markers such as *Muc4* and *Ccl6* following EP300/CREBBP inhibition (Figures 3B,D). This is further supported by the negative correlation with secretory cell gene sets by GSEA (Supplementary Figure 3i). Conversely, this is in line with positive enrichment of Gene Ontology biological process (GO:BP) terms such as smoothened signaling pathway and tissue morphogenesis (Supplementary Figures 3j,k). Irrespective of the exact mechanism, we demonstrate that the paradoxical increase of organoid size after inhibition of EP300/CREBBP bromodomains may be explained by upregulation of *Lgr5, Egr1*, and genes associated with developmental processes, at the cost of IEC differentiation.

### GSK-LSD1 Broadly Affects IEC Composition

**Figure 4:**
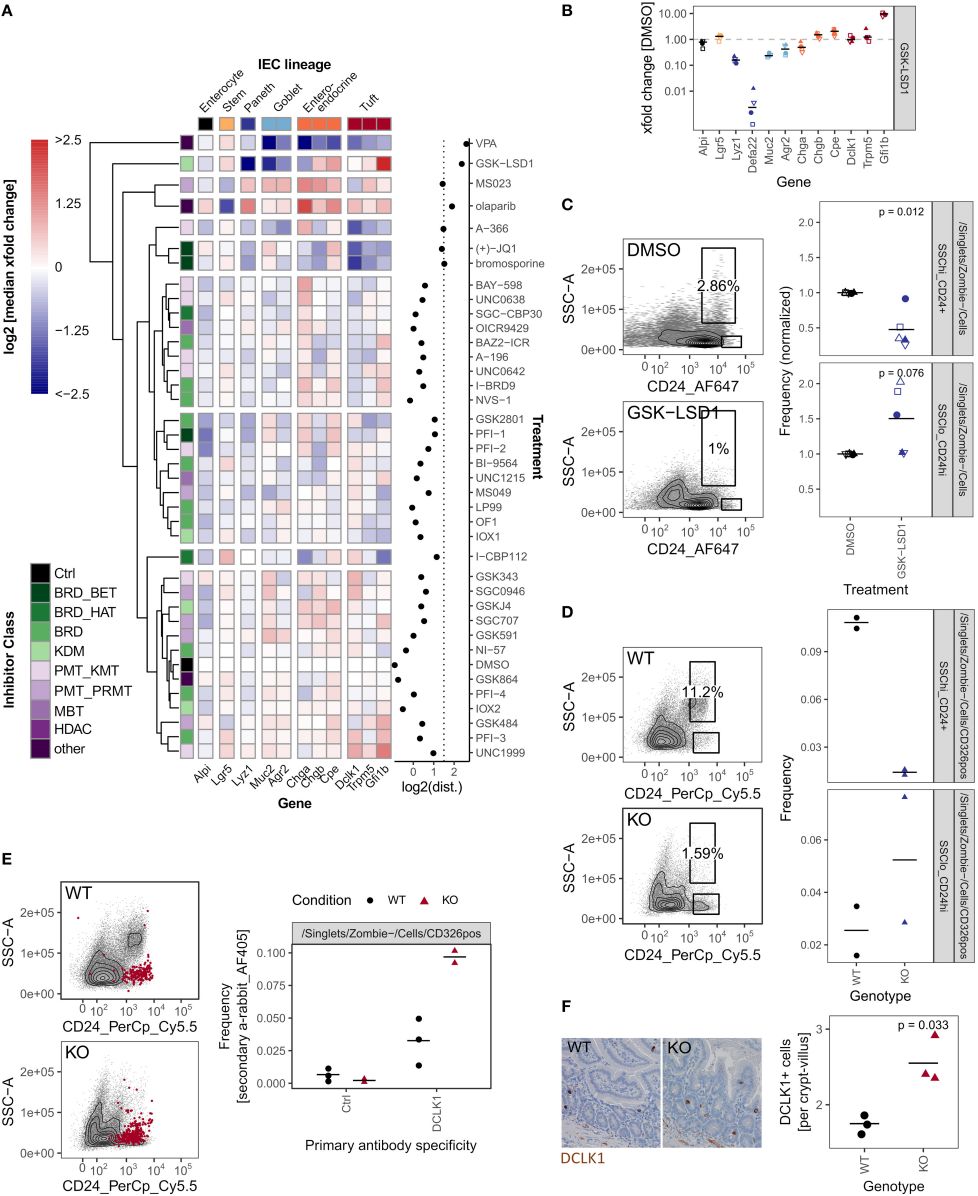
GSK-LSD1 broadly affects IEC composition. **(A)** Gene expression of organoids treated with DMSO or inhibitors for 96 h, measured by qRT-PCR. Color scale represents log2 of median of xfold change relative to DMSO-treated organoids of 4 biol. replicates. IEC lineage marker genes are indicated on x-axis. Inhibitor class is indicated on y-axis. Clustering tree is based on Euclidean distance. Log2 of the Euclidean distance (“perturbation”) is indicated in the right panel, the line at x = 1.5 indicates inhibitors that were followed up in further experiments. Samples treated with HDAC inhibitors and gene *Defa22* were excluded from the analysis. Euclidean distance including *Defa22* is shown in Supplementary Figure 4a. **(B)** Gene expression of organoids treated with GSK-LSD1 for 96 h measured by qRT-PCR. xfold change relative to DMSO-treated organoids. 4 biol. replicates, indicated by shape. Median highlighted. **(C)** Flow cytometry of organoids treated with DMSO or GSK-LSD1 for 96 h. Gating of representative replicates (left) and normalized frequencies of SSC^hi^_CD24+ and SSC^lo^_CD24^hi^ populations of 5 biol. replicates, indicated by shape. Mean highlighted. Paired *t*-test (right). **(D)** Flow cytometry of small intestinal crypts isolated from *Villin*-Cre+ *Lsd1*^fl/fl^ (KO) mice with intestine-specific deletion of *Lsd1* or wild type (WT) littermates. Gating of representative replicates (left) and frequencies of SSC^hi^_CD24+ and SSC^lo^_CD24^hi^ populations of 2/2 mice (right). **(E)** Frequency of DCLK1+ cells measured by intracellular flow cytometry in small intestinal crypts from WT and KO mice. Overlay of positive cells for secondary anti-rabbit staining, representative replicate (left). Quantification of intracellular staining with rabbit anti-DCLK1 primary antibody or control in small intestinal crypts isolated from WT or KO mice. 3/2 mice. Minimum 7,000 viable cells in parent gate (right). **(F)** DCLK1+ cells per crypt-villus pair in duodenum of WT and KO mice. Immunohistochemistry staining of tissue sections. Representative staining (left) and quantification in 3/3 mice, mean highlighted. Unpaired *t*-test (right).

So far, we have used organoid size as a probe selection criteria. Additionally, we performed qRT-PCR on 12 genes associated with specific cell lineages (Haber et al., 2017). We found that, after our positive control VPA, treatment with GSK-LSD1 leads to the largest perturbation of the IEC lineage marker profile as determined by calculating the Euclidean distance of the gene expression xfold changes relative to DMSO treatment (Figure 4A, Supplementary Figures 4a,f–k). Flow cytometry screening of inhibitor treated organoids showed primarily moderate changes in surface marker expressions (Supplementary Figures 4b,c). Although GSK484 and SGC0946 caused the most perturbation in surface marker populations, they showed little effect by qRT-PCR and thus we did not pursue these probes further (Supplementary Figures 4d,e). Treatment with GSK-LSD1 markedly reduced gene expression of Paneth and goblet cell markers, but caused an increase in enteroendocrine and tuft cell marker genes, particularly *Gfi1b* (Figure 4B). This supports our recent work in which we found that Lysine-specific Demethylase 1A (LSD1, KDM1A) is required for Paneth cell differentiation and contributes to goblet cell differentiation (Parmar et al., 2020; Zwiggelaar et al., 2020). Paneth cells are commonly gated as SSC^hi^ CD24+ population in flow cytometry (Sato et al., 2011). In line with a strong reduction of the Paneth cell marker genes *Lyz1* and *Defa22*, we find this population significantly reduced in GSK-LSD1 treated organoids (Figures 4B,C). Furthermore, we observed that the pattern of CD24+ expressing cells in GSK-LSD1 treated organoids differs from control organoids in flow cytometry, with increase of a SSC^lo^_CD24^hi^ population (Figure 4C). This pattern change was even more pronounced in SI crypt IECs from *Villin*-Cre+ *Lsd1*^f/f^ mice, which conditionally lack *Lsd1* in IECs, compared to wild type (WT) littermates (Figure 4D). Similar gating has previously been associated with enteroendocrine cells and their progenitors (Sato et al., 2011), which thus fits with our previous observation that enteroendocrine progenitors such as *Neurod1* and *Neurog3* are upregulated in *Villin*-Cre+ *Lsd1*^f/f^ mice (Zwiggelaar et al., 2020). However, upon performing intracellular flow cytometry staining for the canonical tuft cell marker DCLK1, we found that also a DCLK1^hi^ population fell within this gate and is increased in *Lsd1*-deficient crypts (Figure 4E). In support, there was a modest yet significant increase of DCLK1+ cells in duodenal tissue sections as well as colon sections from *Villin*-Cre+ *Lsd1*^f/f^ mice compared to WT littermates (Figure 4F, Supplementary Figure 4l). Together, this example highlights that the epigenetic probe library contains inhibitors that are able to completely mimic the phenotype that is seen upon genetic deletion *in vivo*.

### BET Inhibition Reduces Relative Abundance of Tuft Cells

**Figure 5:**
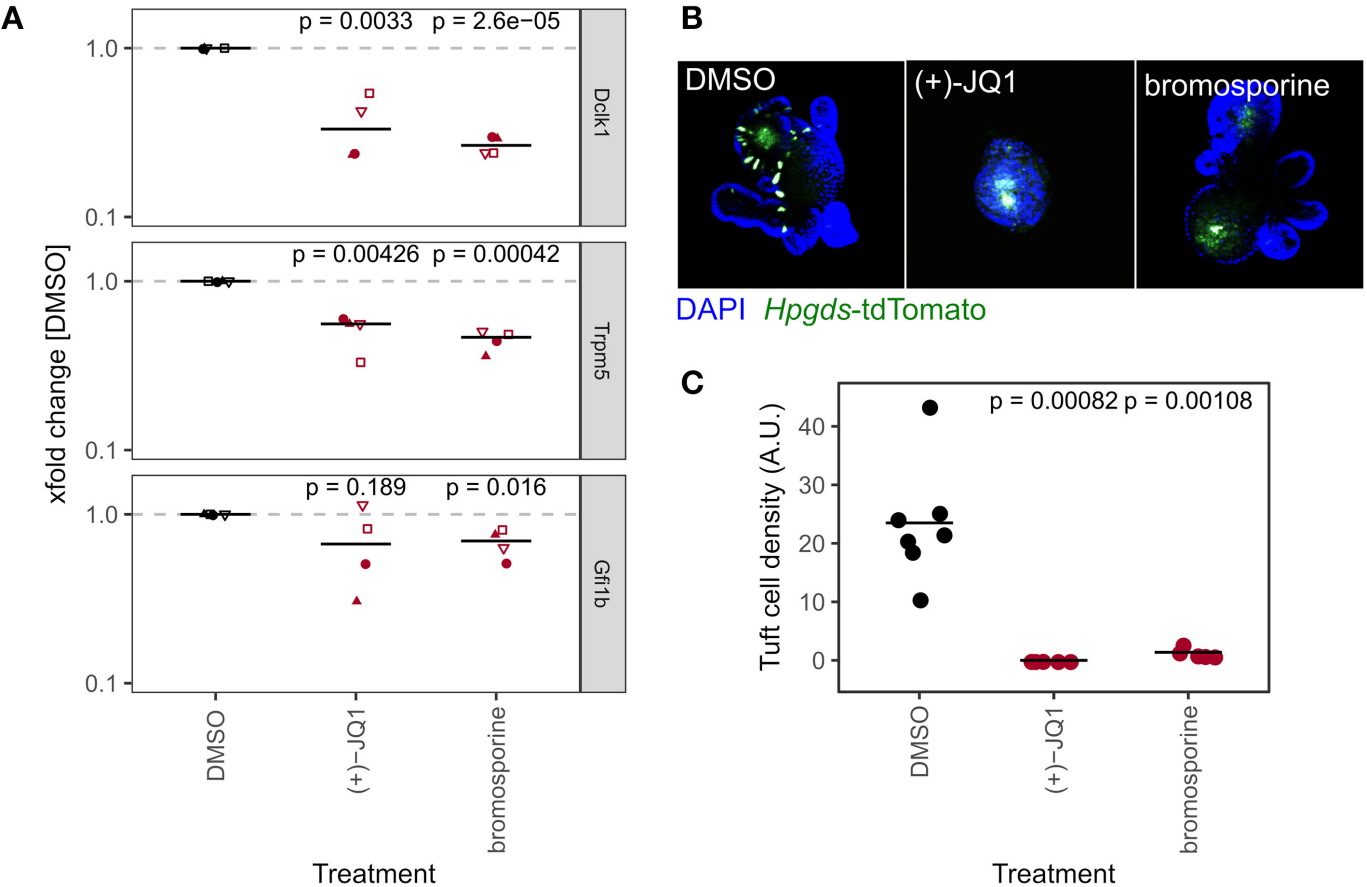
BET inhibition reduces relative abundance of tuft cells. **(A)** Gene expression of tuft cell marker genes of organoids treated with DMSO, (+)-JQ1, or bromosporine for 96 h, measured by qRT-PCR. xfold change relative to DMSO-treated organoids. 4 biol. replicates, indicated by shape. Median highlighted. Paired t-test. **(B)** Representative *Hpgds*-tdTomato tuft cell reporter organoids treated with DMSO, (+)-JQ1, or bromosporine for 8 days with one passage. 10x magnification, max. intensity projection. **(C)** Tuft cell density in *Hpgds*-tdTomato organoids with DMSO, (+)-JQ1, or bromosporine for 8 days. Each dot represents one organoid. Unpaired t-test.

Secretory cell lineage differentiation, such as goblet and Paneth cells, is well-studied and is generally thought to involve NOTCH-mediated lateral inhibition. Tuft cell differentiation, however, is less defined. Therefore, we next focused on the BRD/BET inhibitors (+)-JQ1 and bromosporine in our marker gene expression dataset (Figure 4A) as treatment with these led to a strong reduction of tuft cell marker genes *Dclk1, Trpm5*, and *Gfi1b* (Figure 5A). Organoids treated with these probes also had altered expression in some of the other IEC lineage marker genes, but the downregulation of tuft cell marker genes was consistent and prominent (Supplementary Figure 5a). Although probe A-366, an inhibitor of Euchromatic histone-lysine N-methyltransferase 1 and 2 (EHMT1/2, GLP/G9A) also reduced tuft cell marker genes, two other EHMT1/2 inhibitors, UNC0638 and UNC0642, did not (Figure 4A, Supplementary Figure 4k). (+)-JQ1 inhibits Bromodomain-containing protein 2 (BRD2), BRD3, BRD4, and BRDT while bromosporine is a pan-bromodomain inhibitor. In our hands, these two probes did not affect overall organoid growth in the 96 h course of the screen experiment (Supplementary Figures 2a, 5B), but (+)-JQ1 treatment affected organoid morphology when inhibitor treatment was continued after passaging (Figure 5B). Others have reported that (+)-JQ1 treatment strongly reduced the efficiency of crypts to form organoids (Bolden et al., 2014). Tuft cell quantification after treatment of *Hpgds2*-tdTomato reporter organoids indeed confirmed a complete lack of tuft cell differentiation in organoids treated with either (+)-JQ1 or bromosporine (Figures 5B,C), suggesting that BRD proteins are necessary for the tuft cell lineage. BRD2, BRD3, BRD4, and BRDT are mutual targets of (+)-JQ1 and bromosporine, of which BRDT is not expressed in SI crytps or organoids (Supplementary Figure 5c). Interestingly, the BRD2/4 inhibitor PFI-1 did not cause marked changes in tuft cell marker gene expression in our screen (Supplementary Figure 5d). To investigate the role of specific BRDs in tuft cell differentiation in future studies may be worthwile.

### Inhibition of Type I PRMTs Results in Higher Relative Abundance of Secretory Cells and Prevents Growth of Tumor Organoids

**Figure 6:**
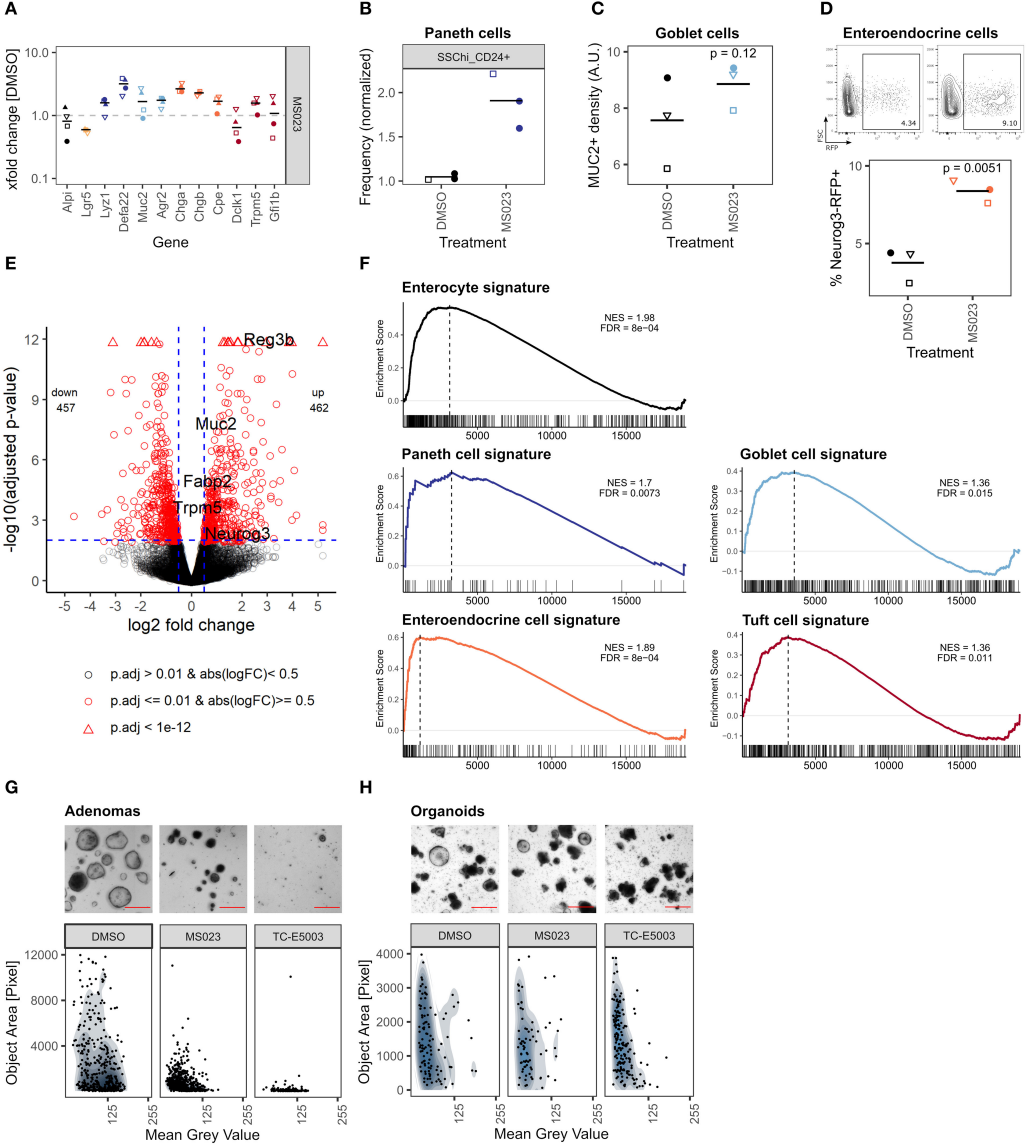
Inhibition of type I PRMTs leads to more mature organoids and prevents adenoma growth. **(A)** Gene expression of organoids treated with MS023 for 96 h measured by qRT-PCR. xfold change relative to DMSO-treated organoids. 4 biol. replicates, indicated by shape. Median highlighted. **(B)** Frequency of SSC^hi^_CD24+ Paneth cells in organoids treated with MS023 for 96 h, normalized to DMSO treatment, measured in flow cytometry screen (Supplementary Figure 4c). 3 wells/2 biol. replicates, indicated by shape. Mean highlighted. **(C)** Density of MUC2+ goblet cells in organoids treated with DMSO or MS023 for 96 h. Median of 3 biol. replicates, indicated by shape. Each value is the median of 4-11 organoids quantified. Paired *t*-test. **(D)** Frequency of enteroendocrine/enteroendocrine progenitor *Neurog3*-RFP+ cells in reporter organoids treated with DMSO or MS023 for 96 h, measured by flow cytometry. Representative gating and quantification in 3 biol. replicates, indicated by shape. Paired *t*-test. **(E)** Volcano plot of mRNA sequencing of untreated vs. MS023 treated organoids, 3 biol replicates per group. Selected genes are highlighted. **(F)** mRNA sequencing of untreated vs. MS023 treated organoids. GSEA for Paneth cell, goblet cell, enteroendocrine cell, and tuft cell signatures from Haber et al. (2017) (GSE92332). Normalized enrichment scores (NES) and false discovery rates (FDR) are indicated. **(G)** *Apc*-deficient adenomas treated with DMSO, MS023, or TC-E5003 for 96 h. Representative well (top, full well shown in Supplementary Figure 6j, scale bar shows 500 μm) and quantification of organoid size and mean gray value, 7 individual wells per condition (bottom). **(H)** Organoids treated with DMSO, MS023, or TC-E5003 for 96 h. Representative replicate (top, full well-shown in Supplementary Figure 6k, scale bar shows 500 μm) and quantification of organoid size and mean gray value, 3 biol. replicates per condition (bottom).

So far, we focused on inhibitors that caused reduced IEC differentiation. However, two probes stood out because they increased the expression of genes associated with Paneth-, goblet-, and enteroendocrine cells (Figure 4A). Of these two, the pan-PARP inhibitor olaparib also had a marked effect on median organoid size and abundance of LGR5+ stem cells (Figures 2C–E). The other probe is MS023, which is an inhibitor of type 1 protein arginine methyltransferases (PRMTs) such as PRMT1, PRMT3, PRMT4 (CARM1), and PRMT8 (Eram et al., 2016) (Figure 6A). Of note, two other PRMT inhibitors in our probe library, SGC707 and MS049 that inhibit PRMT3 and PRMT4/PRMT6, respectively, did not cause similar effects (Supplementary Figure 6a). Although MS023-treated organoids were moderately yet significantly smaller than control organoids and *Lgr5* gene expression was reduced, frequency of *Lgr5*-EGFP stem cells was not significantly affected, and organoids treated for 96 h would renew normally after splitting (Supplementary Figures 6b–d). The upregulation of secretory cell marker genes by the inhibitors was reflected by relative cell abundance of the respective lineages in MS023-treated versus control organoids. SSC^hi^_CD24+ Paneth cells appeared more frequent in MS023 treated organoids in our flow cytometry screen (Figure 6B, Supplementary Figure 4c), and quantification of MUC2+ goblet cells showed a trend in the same direction (Figure 6C). Furthermore, we treated enteroendocrine cell reporter organoids with MS023 and found an increased frequency of *Neurog3*-RFP+ cells compared to the DMSO control (Figure 6D). To get a more detailed overview of how MS023 affects organoids, we performed mRNA sequencing of untreated vs. MS023 treated organoids. We found 462 genes upregulated and 457 genes downregulated with a log2 fold change cutoff of 0.5 and padjust ≤ 0.01 (Figure 6E). Importantly, GSEA of cell-lineage specific gene sets confirmed that MS023-treated organoids have a transcriptome that is enriched for secretory cell lineages (Figure 6F). However, GSEA also indicated an enrichment for genes associated with enterocytes (Figure 6F). Thus, rather than specifically affecting secretory cells, differentiation of all IEC cell lineages seems to be increased in MS023 treated organoids, potentially at the cost of progenitor cells. This is in agreement with positive enrichment of GO:BP terms related to nutrient uptake and response to microbials (Supplementary Figures 6e,f), which are associated with mature enterocytes and Paneth cells, respectively. DNA repair, which is a well established function of type I PRMTs (Guccione and Richard, 2019), was among the negatively correlated GO:BP terms (Supplementary Figures 6e,g). We found that PRMT1 was the type I PRMT with the highest gene expression level in SI crypts and organoids (Supplementary Figure 6h). Enhanced PRMT levels are found in various malignancies and high PRMT1 expression is negatively correlated with survival in colon cancer (Mathioudaki et al., 2008; Jarrold and Davies, 2019). Furthermore, *Prmt1* gene expression was highest in ISC, transit-amplifying cells, and early enterocyte progenitors compared to fully differentiated lineages in a published IEC scRNA-seq dataset (Haber et al., 2017) (Supplementary Figure 6i). We therefore hypothesize that inhibition of type I PRMTs leads to maturation of IECs, which aligns with the observation that differentiated cells have lower *Prmt1* levels. To test if PRMT type I inhibition could hence be used therapeutically to force progenitors, such as those found in WNT-driven tumors, to mature or differentiate, we treated *Apc*-deficient organoids with MS023 or the PRMT1-specific inhibitor TC-E5003. MS023 treated adenomas were smaller and darker than adenomas treated with DMSO control, and TC-E5003 treatment almost completely hindered their growth (Figure 6G, Supplementary Figure 6j). Yet, these probes did not cause growth inhibition of wild type organoids nor did they reduce cell viability (Figure 6H, Supplementary Figures 6k,l). In summary, we show that inhibition of type I PRMT leads to more differentiated organoids and has the potential to hinder proliferation in intestinal tumor organoids, making it an attractive candidate to pursue in future studies.

## Discussion

Working with heterogeneous organoid cultures is challenging with respect to reproducibility and quantification. Our toolkit, which we present in this article enables reproducible results across biological replicates using standard equipment and is suitable for screening setups. We established a quantification workflow that is based on the open source tools ImageJ and Ilastik, which is a simple yet robust alternative to recent stand-alone software options (Borten et al., 2018; Kassis et al., 2019) and could easily be adapted to different tissue organoids. In addition, qRT-PCR and flow cytometry of IEC lineages is sufficiently sensitive for initial screening and was subsequently confirmed by additional methods such as reporter organoids. We used this screening setup to test a set of 39 chemical probes targeting epigenetic modifiers and identified probes that strongly affected organoid size or IEC lineage composition. These new regulators of intestinal epithelial biology are highly interesting candidates for further mechanistic studies.

Probes targeting EP300/CREBBP were designed as cancer therapeutics (Attar and Kurdistani, 2017). Thus, we were surprised to find that inhibition of P300/CREBBP led to an increase of organoid size, which was supported by an expansion of LGR5+ cells and reduction of differentiation (Figure 3). EP300/CREBBP mediate acetylation of histone H3K27 at enhancer elements and promoters, and can act as a transcriptional co-activator with numerous transcription factors (Goodman and Smolik, 2000; Ramos et al., 2010; Wang et al., 2013; Raisner et al., 2018; Weinert et al., 2018). In support of a general activating role for EP300/CREBBP, we found that the majority of genes altered by I-CBP112-treatment were down-regulated (Figure 3D), and many of these genes are established targets of EP300/CREBBP-associated transcription factors. EP300/CREBBP is a well established co-activator of signaling cascades that control cell proliferation, including WNT, NFκB, or MYB signaling. Although we cannot rule out that altering these interactions might contribute to enhanced organoid growth, transcriptional signatures associated with β-Catenin (*Ctnnb1*), NFκB-subunit RelA, or MYB were downregulated after treatment with the EP300/CREBBP inhibitor I-CBP112 (Supplementary Figure 3h). It is difficult to separate the epigenetic modifier (H3K27 acetylation) from the transcriptional co-activator role of EP300/CREBBP, especially since a recent study showed a central role for the bromodomain and HAT domain also for the EP300/CREBBP transcription factor binding capacity (Ortega et al., 2018). Nevertheless, underlining the critical role of the bromodomain, *plt6*-mice that carry a mutation in the EP300KIX domain, which specifically prevents interaction of EP300 with the transcription factor MYB, displayed reduced cell proliferation in the intestine (Sampurno et al., 2013). The EP300/CREBBP bromodomain is critically required for H3K27 acetylation at enhancer elements, a mark of active enhancers, and its inhibition leads to reduced expression of enhancer-proximal genes (Creyghton et al., 2010; Raisner et al., 2018). In the adult small intestine, Sheaffer et al. (2014) described a gain of H3K27Ac at dynamically methylated enhancer sites in differentiated IECs but not LGR5+ ISC. Furthermore, Kazakevych et al. (2017) found that H3K27Ac positive distal elements were a good indicator for cell identity and differentiation status whereas genes positively regulating proliferation were transcribed in most IEC types. EP300/CREBBP has previously been shown to be required for differentiation of embryonic stem cells, muscle cells, and adipocytes (Puri et al., 1997; Zhong and Jin, 2009; Namwanje et al., 2019). In turn, Ebrahimi et al. (2019) recently described that EP300/CREBBP maintains transcription of fibroblast-specific somatic genes and that EP300/CREBBP bromodomain inhibition can promote cellular reprogramming to pluripotency, accompanied by decrease in promoter- and enhancer-associated H3K27 acetylation. We provide evidence that EP300/CREBBP inhibition in the intestinal epithelium can promote proliferation rather than preventing it. It appears plausible that EP300/CREBBP bromodomain activity is critically required to enable transcription of IEC differentiation genes and that in its absence the intestinal epithelium remains immature, accompanied by an enhanced proliferative capacity.

We recently demonstrated a central role of LSD1 in Paneth and goblet cell differentiation and maturation (Parmar et al., 2020; Zwiggelaar et al., 2020). Here, we confirm the critical role of LSD1 for IEC lineage differentiation in an unbiased screen and in addition provide indications that instead of Paneth/goblet cells there is an expansion of DCLK1+ tuft cells that is associated with a CD24^high^_SSC^low^ population by flow cytometry (Figure 4). In contrast, we find that treatment with the BRD/BET inhibitors (+)JQ-1 and bromosporine completely blocks tuft cell differentiation (Figure 5). Tuft cells are important mediators of intestinal type 2 immunity (Gerbe et al., 2016; von Moltke et al., 2016). Our work matches observations of two studies that found that inhibition of the BET bromodomain *in vivo* abolished tuft cells (Bolden et al., 2014; Nakagawa et al., 2016). Using a different BRD/BET probe, Nakagawa et al. (2016) described that the absence of tuft cells was due to blockade of transit-amplifying cells as their intermediate progenitors. While Nakagawa et al. also found a reduction of enteroendocrine cells, another study described an increase of pancreatic NEUROG3+ enteroendocrine progenitors following (+)-JQ1 treatment (Huijbregts et al., 2019). Our findings could be the foundation of using these compounds to modulate immune responses, especially when a type 2 response is unfavorable.

Type I PRMT inhibition with MS023 led to a more differentiated intestinal epithelium without major loss of LGR5+ stem cells (Figure 6, Supplementary Figure 6c). PRMT1 was the most highly expressed type I PRMT and is higher expressed in ISCs and progenitors compared to differentiated cells (Supplementary Figures 6h,i). An evolutionary conserved role of PRMT1 in the adult intestine has been proposed earlier as endogenous PRMT1 knockdown reduces the adult ISC population in *Xenopus* and zebrafish, while transgenic PRMT1 overexpression leads to an increase of ISCs (Matsuda and Shi, 2010; Ishizuya-Oka and Shi, 2011). Furthermore, our observation is very similar to findings of enriched PRMT1 in epidermis progenitors, required for maintenance of this population (Bao et al., 2017). Bao et al. (2017) proposed that PRMT1 is both involved in the maintenance of progenitor/proliferative genes as well as the repression of “differentiation” genes. In agreement with the latter, we found increase of all differentiated IEC lineages after treatment with MS023 (Figures 6A–F). PRMT1 has a wide substrate specificity and mediates both arginine methylation of histones such as H4R3, and non-histone proteins (Huang et al., 2005; Lehman et al., 2020). Elevated PRMT1 expression is found in several cancer types and is associated with poor prognosis and chemoinsensitivity (Altan et al., 2016; Musiani et al., 2020) and pharmacological PRMT inhibitors have recently gained interest as drug candidates for cancer treatment (Guccione and Richard, 2019; Jarrold and Davies, 2019). Targeting cancer stem cells (CSCs) in the gut comes with the challenge that following ablation of LGR5+ CSCs, LGR5- cells have the potential to de-differentiate to CSCs (Morgan et al., 2018). Therefore, forcing differentiation of cancer cells could be an attractive treatment strategy. Indeed, we found that PRMT type I inhibition with MS023 and PRMT1-specific inhibition with TC-E5003 severely impaired growth of *Apc*-deficient tumor organoids but not normal organoids (Figures 6G,H). A conditional *Prmt1*-deficient mouse was recently generated (Choi et al., 2019). Crossing these mice with intestine-specific *Villin*-Cre or tumor-developing *Apc*^min^ mice, could be an elegant way to further study the role of PRMT1 in IEC differentiation and maturation and to investigate the therapeutic potential of PRMT1 inhibition for the treatment of intestinal cancer.

Highly permissive chromatin and transcriptional control of IEC fate, as well as gene regulation by differential chromatin states, have been discussed as opposing models of intestinal epithelial differentiation regulation (Elliott and Kaestner, 2015). Testing a library of highly selective inhibitors targeting more than 20 epigenetic modification enzymes/enzyme families, only two HDAC-inhibitors prevented organoid growth (Figure 2) and the majority of the tested probes did not alter organoid growth or IEC composition. However, we found that few compounds resulted in pronounced changes and these were associated with generally less (EP300/CREBBP, LSD1 inhibition) or more (PRMT type I inhibition) epithelial differentiation. We therefore propose that epigenetic modifiers control the degree of intestinal epithelial differentiation in general, rather than affecting specific cell lineage fate. Whether this parallels the postnatal maturation of the fetal intestinal epithelium remains to be elucidated. Of note, the epigenetic modifiers identified to affect IEC differentiation in our screen share the capacity to both modify histones and to interact with multiple transcription factors. Thus, these molecules could be a key link connecting the epigenetic and the transcriptional layers of gene regulation in the intestinal epithelium. Indeed, work by others supports a model of tightly intertwined epigenetic and transcriptional control and shifting between permissive and dynamic chromatin on a local instead of a global scale. By integrating the investigation of gene expression, open chromatin, and DNA hydroxymethylation in IEC populations with differential expression levels of the transcription factor SOX9, recent elegant work by Raab et al. (2020) identified either highly permissive or dynamic chromatin states at given loci relative to transcription factor binding. EP300 has been described to potentiate SOX9-dependent transcription (Furumatsu et al., 2005) and *Sox9*-deficient intestinal epithelium fails to mature (Bastide et al., 2007). Mapping of EP300-binding sites was recently utilized to identify transcriptional networks in specialized cell types in the placenta (Lee et al., 2019), inspiring further investigation of epigenetic modifier-aided transcription in different IEC lineages.

To summarize, we developed a resource that allows to compare the requirement of various epigenetic modifiers for intestinal epithelial renewal and IEC differentiation. Our results indicate that some epigenetic modifiers with the capacity to both mediate histone modifications and act as transcriptional co-regulators control the balance between an undifferentiated/differentiated epithelial state. Thereby, they lay basis for a fine-tuned transcriptional regulation and rapid adjustment upon injury or pathogenic challenge.

## Methods

### Epigenetic Modifier Inhibitors

The epigenetic modifier inhibitors in the screen experiment were part of the Structural Genomics Consortium Epigenetic Chemical Probes Collection as of March 2016. Probes were reconstituted in DMSO and used at the recommended concentration as listed in Supplementary Table 1. 1 mM valproic acid (VPA) was included as positive control. DMSO vehicle control was matched to the highest concentration used per experiment, maximal 10 μM. PRMT1-specific inhibitor TC-E5003 (Santa Cruz Biotechnology, # sc397056) was included in follow-up experiments and used at 50 μM, equivalent to 10 μM DMSO.

### Mice

C57BL/6JRj wild type (Janvier labs), *Lgr5*-EGFP-IRES-CreERT2 (Jackson Laboratories, stock no: 008875), *Villin*-Cre (el Marjou et al., 2004) (kind gift from Sylvie Robine), *Lsd1*^f/f^ (Kerenyi et al., 2013) (kind gift from Stuart Orkin), and *Apc*^15lox^ (Jackson Laboratories, stock no: 029275) mice were housed under specific-pathogen free conditions at the Comparative Medicine Core Facility (CoMed), Norwegian University of Science and Technology, Norway. For the flow cytometry screening experiment, organoids were generated from C57BL/6 mice housed at the Leibniz-Forschungsinstitut für Molekulare Pharmakologie, Germany. *Hpgds*-tdTomato mice (Bornstein et al., 2018) were housed at University of Montpellier, France. *Neurog3*-RFP mice (Kim et al., 2015) (kind gift from Anne Grapin-Botton) were housed at University of Copenhagen, Denmark. Experiments were performed following the respective legislation on animal protection, were approved by the local governmental animal care committee, and were in accordance with the European Convention for the Protection of Vertebrate Animals used for Experimental and other Scientific purposes.

### Small Intestinal Crypt Isolation

Small intestinal crypts were isolated as described previously (Sato and Clevers, 2012). The proximal half of the small intestine was rinsed, opened longitudinally, cut to small pieces after villi and mucus were scraped off, washed with PBS until the solution was clear, and incubated in 2 mM EDTA/PBS for 30 min at 4°C with gentle rocking. Fragments were subsequently washed with PBS and the crypt fraction was typically collected from wash 2–5. All centrifugation steps were carried out at 300 × g.

### Organoid Culture

Organoids were generated by seeding ca. 250–500 small intestinal crypts in a 50 μl droplet of cold Matrigel (Corning #734-1101) into the middle of a pre-warmed 24-well plate. Matrigel was solidified by incubation at 37°C for 5–15 min and 500 μl culture medium added. Basal culture medium (“ENR”) consisted of advanced DMEM F12 (Gibco) supplemented with 1x Penicillin-Streptomycin (Sigma-Aldrich), 10 mM HEPES, 2 mM Glutamax, 1x B-27 supplement, 1x N2 supplement, (all Gibco) 500 mM N-Acetylcysteine (Sigma-Aldrich), 50 ng/ml recombinant EGF (Thermo Fisher Scientific), 10% conditioned medium from a cell line producing Noggin (kind gift from Hans Clevers), and 20% conditioned medium from a cell line producing R-Spondin-1 (kind gift from Calvin Kuo). ENR culture medium was replaced every 2–3 days. Organoids were passaged at 1:3–1:4 ratio by disruption with rigorous pipetting almost to single cells. Organoid fragments were centrifuged at 300 × g, resuspended in 40–50 μl cold Matrigel per well, and plated on pre-warmed 24-well plates. Organoids derived from different mice or a repetition at least one passage apart are considered biological replicates. Technical replicates, i.e., separate wells, were carried out in some experiments and were pooled for analysis.

### Altering IEC Lineage Composition in Organoids

Protocols to alter the IEC composition in organoids have been described previously (Yin et al., 2014; Kishida et al., 2017; Luu et al., 2018). Organoids were grown for 48 h in ENR or ENR + 3 μM CHIR99021 (Sigma-Aldrich) and 1 mM valproic acid (VPA). Then, media was replaced by ENR, ENR + 3 μM CHIR and 1 mM VPA, ENR + 3 μM CHIR and 10 μM DAPT, or ENR + 10 μM DAPT and 2 μM IWP2. VPA, DAPT, IWP2 were purchased from Cayman Chemicals. Organoids were harvested 72 h after media change.

### Organoid Screen With Epigenetic Chemical Probes Library

Organoids of four biological replicates were passaged to nearly single cells at 1:4 ratio as described above and seeded in 40 μl Matrigel droplets in 24-well plates. 250 μl/well ENR were added immediately after solidification and 250 μl/well ENR + probes at 2x working concentration (see Supplementary Table 1) were added within 30 min. For each biological replicate DMSO vehicle controls were carried out in quadruplicates. Media was replaced after 48 h. Organoids bright-field images were acquired daily on an EVOS2 microscope and after 96 h RNA was harvested.

### Reporter Organoids

*Lgr5*-EGFP organoids were generated as described above from *Lgr5*-EGFP-IRES-CreERT2 mice and maintained for no longer than 3 weeks. Organoids were grown in ENR or ENR + 3μM CHIR99021 (Sigma-Aldrich) as indicated and *Lgr5*-EGFP+ cells were quantified using a BD LSRII flow cytometer (Becton Dickinson) as percentage of viable cells. Tuft cell reporter organoids were generated from *Hpgds*-tdTomato mice (expressing tdTomato under the *Hpgds* promoter) as described above. *Hpgds*-tdTomato+ cells were quantified by confocal microscopy on an Axio Imager Z1 microscope (Zeiss) as number of cells relative to the organoid area after z-stack projection, determined by nuclear staining. Enteroendocrine cell reporter organoids were derived from the proximal small intestine of *Neurog3*-RFP mice (expressing RFP under the *Neurog3* promoter) and cultured as described above using recombinant murine Noggin (100 ng/ml, Peprotech) and 10% R-Spondin conditioned medium. *Neurog3*-RFP+ cells were quantified using a BD FACSAria III flow cytometer (Becton Dickinson) as percentage of viable cells.

### Modified Organoid Growth Conditions With EP300/CREBBP Inhibition

*Lgr5*-EGFP organoids were grown in ENR or ENR + 3 μM CHIR for 96 h. Media was replaced after 48 h. To investigate low growth factor conditions, wild type organoids were grown in ENR or ENR with 1% R-Spondin, ENR with 5 ng/ml EGF, or ENR + 2 μM IWP2 for 192 h. Media was replaced every 48 h.

### Splitting of Organoids After Type I PRMT Inhibition

Organoids were treated with DMSO or MS023 for 96 h, passaged to nearly single cells as described above, and cultured in ENR for additional 96 h. Media was replaced every 48 h.

### Generation of APC-Deficient Adenomas

Eight week old *Apc*^15lox^ × *Lgr5*-EGFP-IRES-CreERT2 mice were administered 2 mg Tamoxifen in corn oil (both Sigma-Aldrich) for 5 consecutive days. Adenomatous polyps developed over the course of a month (ethically approved by the Norwegian Food Safety Authority, FOTS ID: 15888). To generate adenoma organoids, the small intestine was rinsed with PBS, opened longitudinally, polyps were excised, cut into small pieces, and washed in PBS. Next, 5 ml TrypLE express (Thermo Fisher Scientific) was added and incubated for 30 min at 37°C while pipetting every 5–10 min. After incubation, single cells were obtained by passing the supernatant through a 40 μm strainer. Single cells were plated in 50 μl cold Matrigel on a pre-warmed 24-well plate, and cultured in basal culture medium lacking R-Spondin-1 (“EN”). EN culture medium was replaced every 2–3 days.

### Organoid Growth Quantification

Organoid bright-field images were acquired on an EVOS2 microscope (Thermo Fisher Scientific) with 2x magnification. At the starting point of the experiment, for each plate an automation setup was generated to acquire z-stacks with 50 μm spacing either of a single position or 2–4 tiled images covering height and most area of the Matrigel dome for each well. This automation setup was reused at consecutive timepoints. A custom ImageJ/Fiji v1.52n (Schindelin et al., 2012, 2015) macro was used to collect single positions and layers for each well, to save a stack (ImageJ bright-field stack) and projections, and to perform a simple organoid segmentation (“ImageJ workflow”). For the segmentation, a Sobel edge detector was applied to each z-stack layer (ImageJ edge stack), a standard deviation z-projection of the edge stack was generated, and particle analysis with optional manual correction was performed after several binary operations and thresholding. For an improved segmentation that is robust to stitching artifacts, allows to filter out debris and organoid clusters and to distinguish different organoid phenotypes, the ImageJ workflow was combined with the interactive machine learning software Ilastik v1.3.2 (Berg et al., 2019) (“combined workflow”). Training data was taken from the analyzed experiment and excluded from further analysis. In a first step, pixel classification on an intensity summary projection of the ImageJ edge stack was used to separate between background and object outlines. The generated pixel prediction maps were then used as input in a second step of object classification together with minimum projections of the ImageJ bright-field stack. Routinely, the following label classes were used: Organoid, big sphere, small sphere, cluster, debris, background mislabeled as organoid, air bubble, edges of well plate. Objects classified in the latter three object classes were excluded from all timepoints, objects classified as debris or cluster were excluded from 72 to 96 h timepoints. Representative images were arranged using GNU R packages magick and ggimage.

### RNA Isolation, Quantitative RT-PCR, and Analysis

To harvest RNA, organoids in the Matrigel dome were dissolved in 250 μl RNA-solv reagent (Omega Bio-Tek). RNA was isolated using Direct-zol-96 RNA or Direct-zol MiniPrep kit (Zymo Research) according to the manufacturer's instructions, including DNAse digestion. cDNA was transcribed using High-Capacity RNA-to-cDNA Kit (Applied Biosystems) according to the manufacturer's instructions. RNA quality and concentration was assessed on an NanoDrop-1000 instrument (NanoDrop). Samples were handled in 96-well plates and transferred with multichannel pipettes. qRT-PCR was carried out in technical duplicates in 384-well plates on a QuantStudio 5 instrument (Thermo Fisher Scientific) using 2x Perfecta ROX,UNG Fast Mix (Quanta Biosciences) and 5 ng cDNA per reaction in a total volume of 12 μl. Primer-probe combinations were selected based on the Universal Probe Library System (Roche) and are listed in Supplementary Table 2, primers were purchased from Sigma-Aldrich. *Hprt* was used as housekeeping gene. ΔCT values were calculated as ΔCT = CT (housekeeping gene)-CT (gene of interest) (such that higher values indicate higher relative expression); ΔΔCT values referred to the calibrator as indicated, and fold change was calculated as 2^ΔΔCT^. Target gene “perturbation” was calculated as Euclidean distance of the log2 median fold change using GNU R package pheatmap. *Defa22* gene expression was below the detection limit for some samples and was therefore omitted from the Euclidean distance ranking but is provided in the Supplementary Material.

### Flow Cytometry

To obtain single cells, Matrigel in 1–3 wells was disrupted by pipetting, well content was transferred to an Eppendorf tube, centrifuged at 300× g, and supernatant removed. Then, organoids were incubated with 300 μl TrypLE express (Thermo Fisher Scientific) for 37°C for 30 min and pipetted up/down with a 1,000 μl pipet tip prior to and after the incubation. Single cells were stained with Zombie Aqua (Biolegend, 1:1,000 in PBS) for 15 min at room temperature (RT) for live-dead exclusion. If DAPI instead of Zombie Aqua staining was used for live-dead exclusion, it was added it during the last washing step (1:1,000). Samples were incubated with antibody conjugates against CD326-BV605, CD24-PerCp-Cy5.5 or AF647, CD44-BV785, CD117-PE-Cy7 [all Biolegend, see Supplementary Table 3 for detailed list, 1:200 in PBS + 2% fetal calf serum (FCS)], and Ulex Europaeus Agglutinin (UEA)1-Rhodamine (2 μg/ml, Vector Laboratories #RL-1062-2) for 20 min at 4°C. For intracellular staining, samples were subsequently fixed with 2% paraformaldehyde (PFA) for 15min, and incubated with or without rabbit anti-DCLK1 (Abcam #ab31704, 1:500 in PBS/2% FCS/0.05% Saponin) for 1 h at 4°C, followed by incubation with Goat anti-Rabbit IgG-AF405 (Invitrogen, 1:1,000 in PBS + 2% FCS + 0.05% Saponin). Samples were analyzed on a BD LSRII instrument (Becton Dickinson) equipped with 405, 488, 561, 647 nm laser lines. Single fluorochrome stainings of cells and compensation particles (BD CompBead, Becton Dickinson) were included in each experiment. For analysis, FlowJo software v10.6.2 and GNU R/Bioconductor v3.6.3/v3.10 packages flowCore, CytoML/flowWorkspace, ggcyto, flowViz were used (Van et al., 2018). If not indicated otherwise, only samples with more than 10,000 viable cells in the parent gate were included.

### Flow Cytometry Screening

For the flow cytometry screening experiment, organoids were grown in 96 well glass-bottom plates (Cellvis) that were pre-cooled and held on ice during seeding. Organoid fragments in Matrigel (50 μl/well) were distributed in pre-cooled plates with an automated pipette, then plates were transferred to a rotary plate shaker for 30 s at 150 rpm, before the Matrigel was solidified at 37°C. With help of a Viaflo 96-channel pipette (Integra Biosciences) 200 μl/well ENR without or with inhibitors were added of which 100 μl were replaced daily during the 96 h time course. To obtain single cells, culture media was removed, 100 μl/well TrypLE express (Thermo Fisher Scientific) added and Matrigel disrupted by repeated pipetting with a multichannel pipette. Staining with Zombie Aqua, CD326-BV421, CD24 -PerCp-Cy5.5, CD44-AF647, CD117-PE-Cy7 (all Biolegend, see Supplementary Table 3 for detailed list), and UEA1-FITC (Invitrogen) was carried out as described above. Samples were run on a MACSQuant X instrument (Miltenyi Biotec) equipped with 405, 488, 647 nm laser lines and analyzed as described above. Euclidean distance clustering tree of normalized median population frequencies was generated with GNU R package ggtree (Yu et al., 2018).

### Confocal Microscopy and Quantification

For immunofluorescence staining, organoids were grown in 30 μl/well Matrigel droplets in a 8-well microscopy chamber (Ibidi) that was pre-warmed for seeding. After 96 h incubation, the organoids were fixed in 4% paraformaldehyde and 2% sucrose for 30 min at RT, washed, and permeabilized with 0.2% Triton-X100 in PBS. Free aldehyde groups were blocked using 100 mM glycine, followed by blocking buffer (1% BSA, 2% NGS diluted in 0.2% Triton-X100 in PBS) for 1 h at RT. The organoids were incubated overnight at 4°C with a primary antibody against KI67 (Invitrogen #MA5-14520; 1:200) or MUC2 (Santa Cruz #sc-15334; 1:200) in blocking buffer, followed by three washes with slight agitation. Next, the organoids were incubated with Goat anti-Rabbit IgG-AF488 (Invitrogen, 1:500), UEA1-Rhodamine (Vector Laboratories #RL-1062-2, 2 μg/ml), and Hoechst 33342 overnight at 4°C. After washing, the organoids were mounted using Fluoromount G (Thermo Fisher Scientific), and visualized using a LSM880 confocal microscope (Zeiss). UEA1/MUC2-positive cells were manually counted for ≥5 organoids per biological replicate in a middle plane of a z-stack. Cell numbers are reported relative to the area of the z-stack projection of each organoid, determined by nuclear staining.

### Immunohistochemistry and Immunofluorescence Staining of Paraffin-Embedded Tissue and Quantification

Immediately after euthanizing mice, the intestinal tissues were removed, washed with PBS, fixed in 4% formaldehyde for 48–72 h at RT, and embedded in paraffin wax. Staining was carried out on 4 μm paraffin sections. The sections were rehydrated and treated with 3% hydrogen peroxide for 10 min at RT. Antigens were retrieved by boiling the slides in citrate buffer (pH6) in a microwave for 15 min. For immunohistochemistry staining of duodenum sections, the sections were incubated overnight at 4°C with anti-DCLK1 (Abcam #ab31704; 1:1,500) in TBS + 0.025% Tween 20 + 1% BSA. Specific binding was detected with Envision-HRP (Dako) and DAB (Dako) and images were acquired on a EVOS2 microscope (Thermo Fisher Scientific) with 10x magnification. DCLK1+ cells were quantified for ≥30 crypt-villus pairs per mouse. Representative images were acquired on a Eclipse Ci-L microscope (Nikon) with 20x magnification. For immunofluorescence staining of colon sections, slides were blocked with PBS + 1% BSA + 2% goat serum + 0.2% Triton X-100 for 1h at RT and incubated overnight at 4°C with anti-DCLK1 antibody (Abcam #ab31704; 1:250) in PBS + 1% BSA +1% goat serum + 0.05% Tween 20. Specific binding was detected with Goat anti-Rabbit IgG-AF488 (Invitrogen, 1:1,000) for 1h at 37°C while nuclei were stained with DAPI (1:1,000). Slides were mounted with Fluoromount G (Thermo Fisher Scientific) and images were acquired on a LSM880 confocal microscope (Zeiss) with 20x magnification. DCLK1+ cells were quantified for ≥50 crypts per mouse.

### mRNA Sequencing

Organoid RNA was isolated and treated with DNAse with Quick-RNA Micro prep kit (Zymo Research) according to manufacturer's instructions. RNA integrity numbers were found to be ≥7. For the I-CBP112 inhibitor study, library preparation was done using the Illumina TruSeq Stranded protocol. Library concentrations were quantified with the Qubit Fluorometric Quantitation system (Life Technologies) and the size distribution was assessed using a 2100 Bioanalyzer automated electrophoresis system (Agilent). For sequencing, samples were diluted and pooled into NGS libraries in equimolar amounts and sequenced at 75 bp single-read chemistry on an Illumina NS500 MO flow-cell on a Ilumina NextSeq 500 instrument (Illumina) by the Genomics core facility (GCF, NTNU). For the MS023 study, library preparation was done using the NEB Next Ultra RNA Library Prep Kit with poly(A) mRNA enrichment and samples were sequenced at 150X2 bp paired-end chemistry on a Illumina NovaSeq 6000 instrument by Novogene (UK) Co.

### mRNA Sequencing Analysis

Read quality was assessed using FastQC v0.11.8, reads were aligned with STAR v2.7.3a to the Mus musculus genome build mm10, and MultiQC v1.7 was used to summarize logs from STAR and FastQC (Dobin et al., 2013; Leggett et al., 2013; Ewels et al., 2016). The number of reads that uniquely aligned to the exon region of each gene in GENCODE annotation M18 of the mouse genome was then counted using featureCounts v1.6.4 (Liao et al., 2014; Frankish et al., 2019). Genes that had a total count <10 were filtered out. Differential expression was then determined with GNU R/Bioconductor v3.6.1/v3.10 package DESeq2 v1.26.0 using default settings and shrunken log2foldchange was calculated with the apeglm method (Love et al., 2014; Zhu et al., 2019). GSEA enrichment was performed using GNU R/Bioconductor v3.6.3/v3.10 package ClusterProfiler v3.14.3 by shrunken log2 fold change and with the shrunken log2 fold change as weights using 10,000 permutations (Yu et al., 2012). Gensets for celltype signatures were assembled based on single-cell and bulk RNA-Sequencing data from sorted samples based on datasets by Haber et al. (2017) (GSE92332) and Muñoz et al. (2012) (GSE33949). Transcription factors interacting with murine or human EP300 or CREBBP were retrieved from protein-protein interactions with an minimum medium experimental confidence level (≥0.4) from STRING-DB v11 (Szklarczyk et al., 2019). Genesets regulated for the mouse and human version of these transcription factors were retrieved from TRRUST v2 (Han et al., 2018). For human genesets, murine orthologue genes retrieved from Ensembl GRCh38.p13 through GNU R/Bioconductor v3.6.3/v3.10 package biomaRt v2.42.1 (Durinck et al., 2005) were used for enrichment. Genesets for characterization of Biological Process were directly obtained from The Gene Ontology Consortium (2019).

### Data Processing and Statistical Analysis

Data was processed and statistical analysis was carried out with GNU R v3.6.3 using the packages tidyverse and ggpubr (Wickham et al., 2019). Pearson correlation coefficient, and paired or unpaired t-test were calculated as indicated, assuming normal distribution. Median or mean are shown as indicated. In boxplots, the box represent the 25, 50, and 75% percentiles and whiskers represent 1.5 × IQR.

## Code Availability

The ImageJ script used for organoid segmentation is available at: https://github.com/jennyostrop/Fiji_organoid_brightfield_processing and deposited under https://doi.org/10.5281/zenodo.3951126.

## Data Availability Statement

The Imaging data from the initial screen was deposited to the Image Data Resource (Williams et al., 2017; https://idr.openmicroscopy.org) under accession number idr0092. The Ilastik projects and respective training data of the initial screen organoid segmentation were deposited to Zenodo under https://doi.org/10.5281/zenodo.4311473. The qRT-PCR data from the initial screen was deposited along with processed data from follow-up experiments to BioStudies database at EMBL-EBI (Sarkans et al., 2018; https://www.ebi.ac.uk/biostudies) under accession number S-BSST447. RNA-seq data were deposited in the ArrayExpress database at EMBL-EBI (Athar et al., 2019; https://www.ebi.ac.uk/arrayexpress) under accession number E-MTAB-9290 (I-CBP112-treated samples) and E-MTAB-9291 (MS023-treated samples).

## Ethics Statement

The animal study was reviewed and approved by the Norwegian Food Safety Authority (FOTS ID: 15888).

## Author Contributions

JO and MO designed the study. JO, RZ, MT, FG, HL, AD-S, NP, SR, and MO performed the experiments. JO, RZ, MT, FG, KB, and HL analyzed the data. JO, KB, and HL curated the data. CA provided critical materials. PJ, KJ, CA, and MO supervised or provided critical insight. JO, RZ, and MO wrote the manuscript with subsequent input from other authors. All authors contributed to the article and approved the submitted version.

## Conflict of Interest

The authors declare that the research was conducted in the absence of any commercial or financial relationships that could be construed as a potential conflict of interest.

## Supplementary Material

Supplementary Figure 1 **(a)** Organoid growth 24-96h, representative well from experiment quantified in Figure 1C. The area shown in Figure 1A is marked. **(b)** Organoid segmentation with ImageJ/Fiji (“IJ,” left) and combined ImageJ/Fiji and Ilastik (“Ilastik,” right) workflow, 48 and 96 h timepoints. The ImageJ segmentation sufficiently identifies objects while the combined workflow is more accurate and can distinguish phenotypes such as organoid, sphere, cluster, or debris. **(c)** Quantification result with ImageJ workflow. Pooled data from 2 biol. replicates. **(d)** Flow cytometry gating strategy. **(e)** Flow cytometry of organoids grown for 48 and 96 h. Population frequencies of flow cytometry staining in Cells, Cells/CD326^*hi*^ and Cells/CD24+ parent gate, 3 biol. replicates, indicated by shape, mean highlighted (bottom). Log2 fold change normalized to 96 h timepoint, visualized as dot plot, dot size corresponds to absolute log2 fold change (top). **(f)** Flow cytometry of organoids cultured for 48 h followed by 72 h (48_72 h) with normal culture medium (ENR_ENR), or culture medium containing CHIR+VPA_CHIR+VPA, CHIR+VPA_CHIR+DAPT, or CHIR+VPA_IWP2+DAPT to modify IEC composition. Population frequencies in Cells, Cells/CD326^*hi*^ and Cells/CD24+ parent gate. Log2 fold change normalized to ENR_ENR treatment. 3 biol. replicates, indicated by shape, mean highlighted (bottom) and visualized as dot plot, dot size corresponds to absolute log2 fold change (top). **(g)** Flow cytometry of organoids cultured for 48 h followed by 72 h (48_72 h) as indicated. Staining of representative replicate, fluorescence intensity median and q25 and q75 percentile. Cell count in Cells parent gate is indicated. **(h)** Flow cytometry of organoids cultured for 48 h followed by 72 h (48_72 h) as indicated. SSC vs. CD24_PerCp-Cy5.5 staining in Cells parent gate, frequency in SSC^hi^_CD24+ gate is indicated.

Supplementary Figure 2 **(a)** Segmentation results of combined ImageJ/Ilastik (left) and ImageJ (right) workow of organoids treated with DMSO or inhibitors for 0–96 h. 4 biol. replicates. **(b)** Median organoid area and relative *Lgr5* gene expression in DMSO or olaparib-treated organoids. 4 biol. replicates, indicated by shape. Median highlighted. Paired *t*-test. **(c)** Median organoid area and relative *Lgr5* gene expression in DMSO, CI-994, or LAQ824-treated organoids. 4 biol. replicates. Median highlighted. Paired *t*-test. **(d)** Frequency of *Lgr5*-EGFP stem cells in reporter organoids treated with DMSO or olaparib, measured by flow cytometry. Gating of representative replicate (top) and percentage of GFP+ cells normalized to DMSO condition of 5 biol. replicates, indicated by shape. Mean highlighted. Paired *t*-test (bottom). Minimum 5,000 viable cells in parent gate.

Supplementary Figure 3 **(a)** Median organoid area and relative *Lgr5* gene expression in DMSO, SCG-CBP30, or I-CBP112 treated organoids. 4 biol. replicates, indicated by shape. Median highlighted. Paired *t*-test. **(b)** Organoids treated with DMSO, SGC-CBP30, or I-CBP112 for 96 h. Percentage of organoids classified as big spheres in combined ImageJ/Ilastik segmentation workflow. 4 biol. replicates, indicated by shape. Mean highlighted. Paired *t*-test. **(c)** Confocal microscopy of DMSO, SCG-CBP30, or I-CBP112 treated organoids. 10x magnification, max. intensity projection. Full well, the organoids shown in Figure 2B are marked. **(d)** Frequency of *Lgr5*-EGFP stem cells in reporter organoids grown in ENR or ENR + CHIR and treated with DMSO, SCG-CBP30, or I-CBP112, measured by flow cytometry. Gating of representative replicate (top) and percentage of GFP+ cells normalized to ENR DMSO condition of 3 biol. replicates, indicated by shape. Mean highlighted. Paired *t*-test (bottom). **(e)** Representative replicate of *Lgr5*-EGFP reporter organoids grown in ENR or ENR + CHIR and treated with DMSO, SCG-CBP30, or I-CBP112 for 96 h. **(f)** Object area of organoids grown in ENR, ENR + low R-Spondin (1%), ENR + low EGF (1 ng/ml), or ENR + IWP2 (Wnt pathway inhibitor) for 192 h, treated with DMSO, SGC-CBP30, or I-CBP112. 3 biol. replicates, indicated by shape. **(g)** Representative Replicate of organoids grown in ENR, or low growth factor conditions for 192 h, treated with DMSO, SGC-CBP30, or I-CBP112. **(h)** mRNA sequencing of untreated vs. I-CBP112 treated organoids. GSEA of target genes of the convergence of murine and human transcription factors from the TRRUST transcriptional interactions database and proteins interacting with murine or human EP300 or CREBBP with a minimum medium experimental confidence level (≥0.4) from the STRING protein-protein interactions database. Enriched transcription factor target gene sets with a *p*-value ≥0.1 are shown. All normalized enrichment score (NES) values are indicated. **(i)** mRNA sequencing of untreated vs. I-CBP112 treated organoids. GSEA for enterocyte, Paneth cell, goblet cell, enteroen-docrine cell, tuft cell signatures from Haber et al. (GSE92332). **(j)** GSEA for Gene Ontology biological process (GO:BP) terms. Normalized enrichment score (NES) is indicated by color. Top 50 categories are shown. **(k)** GSEA for Gene Ontology term “smoothened signaling pathway” (GO:0007224, NES = 2.0406), and “tissue morphogenesis” (GO:0048729, NES = 1.5148).

Supplementary Figure 4 **(a)** Log2 of the Euclidean distance (“perturbation”) of IEC lineage marker gene expression of organoids treated with DMSO or inhibitors for 96 h, including gene *Defa22*. Samples treated with HDAC inhibitors were excluded from the analysis. Order is the same as in Figure 4A. **(b)** Scheme of flow cytometry screen. Organoids were cultivated in 96-well plates, culture media was changed daily. All pipetting steps were carried out with automated pipettes or multichannel pipettes and samples were acquired on a flow cytometer with automated plate handling. **(c)** Flow cytometry screening of organoids, treated with DMSO or inhibitors for 96 h. Population frequencies in Cells, FSC_CD326^*hi*^ and FSC_CD24+ parent gate. Log2 fold change of gated populations normalized to DMSO treatment. Median of 4wells/3 biol. replicates, samples with ≤4,000 cells and populations containing ≤40 cells were excluded. Dot size corresponds to absolute log2 fold change. Tree is based on Euclidean distance clustering of populations in Cells parent gate. **(d)** Gene expression of organoids treated with DMSO or GSK484 for 96 h, measured by qRT-PCR. GSK484 is an inhibitor of arginine deiminase (PADI4). 4 biol. replicates, indicated by shape. Median highlighted. **(e)** Gene expression of organoids treated with DMSO or SGC0946 for 96 h, measured by qRT-PCR. SGC0946 is an inhibitor of H3 lysine-79 specific Histone-lysine N-methyltransferase (DOT1L). 4 biol. replicates, indicated by shape. Median highlighted. **(f–k)** Gene expression of *Alpi, Lgr5, Lyz1, Defa22, Muc2, Agr2, Chga, Chgb, Cpe, Dclk1, Trpm5 Gfi1b* in organoids treated with DMSO or inhibitors for 96 h, measured by qRT-PCR. xfold change relative to DMSO-treated organoids. 4 biol. replicates, indicated by shape. Median highlighted. Paired *t*-test and false discovery rate (FDR) shown for *p*-values ≤0.1. **(l)** DCLK1+ cells per crypt in colon of Villin-Cre+ Lsd1^*f*/*f*^ (KO) mice with intestine-specific deletion of *Lsd1* and wild type (WT) littermates. Immunofluorescence staining of tissue sections. Representative staining (left) and quantification in 5/3 mice, mean highlighted. Unpaired *t*-test (right).

Supplementary Figure 5 **(a)** Gene expression of organoids treated with (+)-JQ1 (left) or bromosporine (right) for 96 h, measured by qRT-PCR. xfold change relative to DMSO-treated organoids. 4 biol. replicates, indicated by shape. Median highlighted. **(b)** Organoids treated with (+)-JQ1 or bromosporine for 96 h. Representative replicate from screen experiment. **(c)** BRD transcripts per million (tpm) values in mRNA sequencing datasets of small intestinal crypts (3 mice) and organoids (4 biol. replicates) from Zwiggelaar et al. (2020) (control groups from E-MTAB-9077, E-MTAB-7862). **(d)** Gene expression of organoids treated with PFI-1 for 96 h, measured by qRT-PCR. PFI-1 is an inhibitor of BRD2/BRD4. xfold change relative to DMSO-treated organoids. 4 biol. replicates, indicated by shape. Median highlighted.

Supplementary Figure 6 **(a)** Gene expression of organoids treated with SGC707 (left) or MS049 (right) for 96 h, measured by qRT-PCR. xfold change relative to DMSO-treated organoids. 4 biol. replicates, indicated by shape. Median highlighted. **(b)** Median organoid area and relative *Lgr5* gene expression in DMSO or MS023-treated organoids. 4 biol. replicates, indicated by shape. Median highlighted. Paired *t*-test. **(c)** Frequency of *Lgr5*-EGFP stem cells in reporter organoids treated with DMSO or MS023 for 96 h, measured by flow cytometry. 5 biol. replicates, indicated by shape. Mean highlighted (bottom). Paired *t*-test. **(d)** Organoids treated with DMSO or MS023 for 96 h, and passaged to ENR for additional 96 h. Images taken at 96 h timepoint and 24 and 96 h after passaging. **(e)** mRNA sequencing of untreated vs. MS023 treated organoids. GSEA for Gene Ontology biological process (GO:BP) terms. Normalized enrichment score (NES) is indicated by color. Top 50 categories are shown. **(f)** mRNA sequencing of untreated vs. MS023 treated organoids. GSEA for GO:BP terms “positive regulation of cell development” (GO:0010720, NES = 1.6325), “lipid digestion” (GO:0044241, NES = 1.9547), and “response to bacterium” (GO:0009617, NES = 1.9257). **(g)** mRNA sequencing of untreated vs. MS023 treated organoids. GSEA for GO:BP term “DNA repair” (GO:0006281, NES = −1.5531). **(h)** PRMT transcripts per million (tpm) values in mRNA sequencing datasets of small intestinal crypts (3 biol. replicates) and organoids (4 biol. replicates) from Zwiggelaar et al. (2020) (control groups from E-MTAB-9077, E-MTAB-7862). **(i)** Box plots showing tpm values from plate based scRNA sequencing from Haber et al. (GSE92332). Tuft cell clusters have been merged into one group. **(j)** *Apc*-deficient adenomas treated with DMSO, MS023, or TC-E5003 for 96 h. Representative well. **(k)** Organoids treated with DMSO, MS023, or TC-E5003 for 96 h. Representative replicate. **(l)** Viability of organoids treated with DMSO, MS023, or TC-E5003 for 96 h, measured by flow cytometry as frequency of Zombie Aqua-negative cells in Singlet parent gate. 3 biol. replicates, indicated by shape.

Supplementary Table 1 Inhibitors and concentrations used.

Supplementary Table 2 qRT-PCR primers and probes used.

Supplementary Table 3 Materials, reagents, and software used.
