# Supplementary material for: A Semi-automated Organoid Screening Method Demonstrates Epigenetic Control of Intestinal Epithelial Differentiation
Source: Front Cell Dev Biol. 2021 Jan 21;8:618552. doi: 10.3389/fcell.2020.618552 (PMC7872100; doi:10.3389/fcell.2020.618552)
Supplement: Supplementary Table 1 — Inhibitors and concentrations used. [file Data_Sheet_2.pdf]

Table 1:
Inhibitors

Source	ProbeName	InternalProbeID	ProbeSynonyme	PubChem_CID	ChEMBL	ChEBI	InChIKey	InhibitorClass	Target	TargetSynonyme	StockConc [mM]	WorkingConc [µM]
Sigma Aldrich	DMSO			679	CHEMBL504	CHEBI:28262	IAZDPXIOMUYVGZ-UHFFFAOYSA-N	Ctrl			10	10
Cayman Chemicals	VPA			3121	CHEMBL109	CHEBI:83766	NIJJYAXOARWZEE-UHFFFAOYSA-N	Ctrl			100	1000
SGC	(+)-JQ1	1		46907787	CHEMBL1957266	CHEBI:137113	DNVXATUJJDPFDM-KRWDZBQOSA-N	BRD_BET	BRD2, BRD3, BRD4, BRDT (BET)		10	0.2
SGC	bromosporine	7		72943187	CHEMBL3133807	CHEBI:95080	UYBRROMMFMPJAN-UHFFFAOYSA-N	BRD_BET	pan-Bromodomain		10	1
SGC	PFI-1	10	PF-6405761	71271629	CHEMBL2179387		TXZPMHLMPKIUGK-UHFFFAOYSA-N	BRD_BET	BRD2, BRD4		10	1
SGC	SGC-CBP30	16		72201027	CHEMBL3622373	CHEBI:95079	GEPYBHCJBORHCE-SFHVURJKSA-N	BRD_HAT	CREBBP, EP300	CBP, P300	10	1
SGC	I-CBP112	28		90488984	CHEMBL3774655		YKNAKDFZAWQEEO-IBGZPJMESA-N	BRD_HAT	CREBBP, EP300	CBP, P300	10	3
SGC	BI-9564	6		117072549	CHEMBL3823101		BJFSUDWKXGMUKA-UHFFFAOYSA-N	BRD	BRD9, BRD7		10	1
SGC	I-BRD9	27	GSK602	91668541	CHEMBL3769507		WRUWGLUCNBMGPS-UHFFFAOYSA-N	BRD	BRD9		10	1
SGC	LP99	32		91827372	CHEMBL3753082		LVDRREOUMKACNJ-BKMJKUGQSA-N	BRD	BRD9, BRD7		10	1
SGC	PFI-4	13		40642506	CHEMBL3356143		QCIJLRJBZDBVDB-UHFFFAOYSA-N	BRD	BRPF1B		10	1
SGC	NI-57	35		91827373	CHEMBL3752151		UEMQPCYDWCSVCU-UHFFFAOYSA-N	BRD	BRPF1, BRPF2, BRPF3		10	1
SGC	OF1	37		35397514	CHEMBL3770173		YUNQZQREIHWDQT-UHFFFAOYSA-N	BRD	BRPF1B, BRPF2		10	5
SGC	BAZ2-ICR	5		91654625	CHEMBL4296718		RRZVGDGTWNQAPW-UHFFFAOYSA-N	BRD	BAZ2A, BAZ2B		10	1
SGC	GSK2801	9		73010930	CHEMBL3739699		KHWCPNJRJCNVRI-UHFFFAOYSA-N	BRD	BAZ2A, BAZ2B		10	3
SGC	PFI-3	12		78243717	CHEMBL3752911		INAICWLVUAKEPB-QSTFCLMHSA-N	BRD	SMARCA,PB1		10	1
SGC	NVS-1	36		117072550	CHEMBL3785432	CHEBI:149519	XVECNLUKQDKOST-UHFFFAOYSA-N	BRD	CECR2		10	1
SGC	GSKJ4	25		71729975	CHEMBL3183531		WBKCKEHGXNWYMO-UHFFFAOYSA-N	KDM	JMJD3	KDM6B	10	5
SGC	IOX2	30		54685215	CHEMBL3186774	CHEBI:95077	CAOSCCRYLYQBES-UHFFFAOYSA-N	KDM	HIF-1α prolyl hydroxylase-2 (PHD2), JMJD2, JMJD3, FIH	HPH1, EGLN2, KMD4A	10	1
SGC	GSK-LSD1	26		71522234	CHEMBL4301645	CHEBI:95087	BASFYRLYJAZPPL-UONOGXRCSA-N	KDM	LSD1	KDM1A	10	1
SGC	IOX1	29		459617	CHEMBL1230640		JGRPKOGHYBAVMW-UHFFFAOYSA-N	KDM	2OG oxygenases, including the JmjC demethylases		10	10
SGC	UNC1999	20		72551585	CHEMBL3414619	CHEBI:93239	DPJNKUOXBZSZAI-UHFFFAOYSA-N	PMT_KMT	EZH2		10	3
SGC	GSK343	21		71268957	CHEMBL2204995		ULNXAWLQFZMIHX-UHFFFAOYSA-N	PMT_KMT	EZH2		10	3
SGC	OICR9429	38		91623360	CHEMBL3798846	CHEBI:95086	DJOVLOYCGXNVPI-UHFFFAOYSA-N	PMT_KMT	WDR5	MLL1	10	3
SGC	A-366	3		76285486	CHEMBL3109630		BKCDJTRMYWSXMC-UHFFFAOYSA-N	PMT_KMT	EHMT1, EHMT2	G9a, GLP	10	1
SGC	UNC0638	17		46224516	CHEMBL1231795		QOECJCJVIMVJGX-UHFFFAOYSA-N	PMT_KMT	EHMT1, EHMT2	G9a, GLP	10	1
SGC	UNC0642	18		53315878	CHEMBL2441082	CHEBI:95074	RNAMYOYQYRYFQY-UHFFFAOYSA-N	PMT_KMT	EHMT1, EHMT2	G9a, GLP	10	1
SGC	PFI-2	11		71300326	CHEMBL3414622		JCKGSPAAPQRPBW-OAQYLSRUSA-N	PMT_KMT	SETD7		10	1
SGC	A-196	2		117072548			ABGOSOMRWSYAOB-UHFFFAOYSA-N	PMT_KMT	SUV420H1/H2		10	1
SGC	BAY-598	4		117072551	CHEMBL3818617		OTTJIRVZJJGFTK-SFHVURJKSA-N	PMT_KMT	SMYD2		10	1
SGC	MS023	33		92136227	CHEMBL3901808		FMTVWAGUJRUAKE-UHFFFAOYSA-N	PMT_PRMT	PRMT1, PRMT3, PRMT4, PRMT6, PRMT8		10	5
SGC	SGC707	15		90642938	CHEMBL4072005		DMIDPTCQPIJYFE-UHFFFAOYSA-N	PMT_PRMT	PRMT3		10	1
SGC	MS049	34		53868701	CHEMBL3961701		HBOJWAYLSJLULG-UHFFFAOYSA-N	PMT_PRMT	PRMT4, PRMT6		10	5
SGC	GSK591	23		117072552			TWKYXZSXXXKKJU-FQEVSTJZSA-N	PMT_PRMT	PRMT5		10	1
SGC	SGC0946	14		56962337	CHEMBL3087498		IQCKJUKAQJINMK-HUBRGWSESA-N	PMT_PRMT	DOT1L		10	1
SGC	GSK484	22		86340151			MULKOGJHUZTANI-ADMBKAPUSA-N	PMT_PRMT	PAD-4	PADI4	10	10
SGC	UNC1215	19		57339144	CHEMBL2426364		PQOOIERVZAXHBP-UHFFFAOYSA-N	MBT	L3MBTL3		10	1
SGC	CI-994	8	N-acetyldinaline, Tacedinaline	2746	CHEMBL235191		VAZAPHZUAVEOMC-UHFFFAOYSA-N	HDAC	HDAC1, HDAC2, HDAC3, HDAC8		10	10
SGC	LAQ824	31	Dacinostat	6445533	CHEMBL356066	CHEBI:90195	BWDQBBCUWLSASG-MDZDMXLPSA-N	HDAC	HDAC		10	1
SGC	GSK864	24		91864701		CHEBI:94063	DUCNNEYLFOQFSW-PMERELPUSA-N	other	Mutant isocitrate dehydrogenase 1	IDH1	10	0.3
SGC	olaparib	39	AZD2281, KU0059436	23725625	CHEMBL521686		FDLYAMZZIXQODN-UHFFFAOYSA-N	other	PARP		10	1
SantaCruz	TC-E5003			87052	CHEMBL1797447	CHEBI:39867	SHRCVZJKZJGIHQ-UHFFFAOYSA-N	follow-up	PRMT1		50	50
