# Supplementary material for: A Semi-automated Organoid Screening Method Demonstrates Epigenetic Control of Intestinal Epithelial Differentiation
Source: Front Cell Dev Biol. 2021 Jan 21;8:618552. doi: 10.3389/fcell.2020.618552 (PMC7872100; doi:10.3389/fcell.2020.618552)
Supplement: Supplementary Table 2 — qRT-PCR primers and probes used. [file Data_Sheet_3.pdf]

Table 2:
qRT-PCR primers & probes

Gene	IEC_lineage	Used_in_screen	Primer_fwd	Primer_rev	Probe	Product_length
Hprt	no	yes	cctcctcagaccgcttttt	aacctggttcatcatcgctaa	UPL_#95	91
Alpi	Enterocyte	yes	aggatccatctgtcctttggt	ttcagctgccttcttgttcc	UPL_#33	75
Lgr5	Stem	yes	cttcactcggtgcagtgct	gatcagccagctaccaaatagg	UPL_#60	75
Defa21	Paneth	no	gagcagccaggggaagat	cgatttctacaaaggcagatca	UPL_#64	110
Defa22	Paneth	yes	gatgaagagactaatactgaggagca	cgttttctacaaaggcagatca	UPL_#64	131
Defa29	Paneth	no	tgaagagactaaaactgaggagca	caccacggaaaccttctttg	UPL_#6	99
Lyz1	Paneth	yes	ggcaaaaccccaagatctaa	tctctcaccaccctctttgc	UPL_#46	104
Mptx2	Paneth	no	tccaagaagagcacaatgga	aaatgcctttcccttcatgtc	UPL_#105	99
Agr2	Goblet	yes	cctcaacctggtctatgaaaca	accgtcagggatgggtcta	UPL_#2	93
Ccl9	Goblet	no	caagggcttgaaattgaaatg	tcttgaacactgaatccgtgag	UPL_#27	90
Muc2	Goblet	yes	cacgagacccaggaagtacag	gcaaagccactaactgcttgt	UPL_#105	88
Chga	Enteroendocrine	yes	aacttcaagacctggctctcc	ctcaaagctgctgtgttgct	UPL_#33	131
Chgb	Enteroendocrine	yes	cctctcaaatgccctatcca	cacctttgacctcttttccact	UPL_#47	92
Cpe	Enteroendocrine	yes	ccggaagagactctcaaaagc	cggacaaaccctttaacacc	UPL_#105	95
Gfr3a	Enteroendocrine	no	tgatcctgctactggtgctg	ctctgtggcaagggagtttc	UPL_#5	65
Dclk1	Tuft	yes	agaaggcacagcttgcaga	gttattgtggcaggaatctgg	UPL_#25	74
Gfi1b	Tuft	yes	gttgctgaaccagagccttc	ttggggtgtcacgagagg	UPL_#45	64
Lrmp	Tuft	no	gctgcttatggagactacacga	tccgttttctgtggttctga	UPL_#55	90
Pou2f3	Tuft	no	caaagcagcaatgaactcctc	tgattccaacggtaccatga	UPL_#78	69
Trpm5	Tuft	yes	ttctagatggtgtccaccaaaa	gcagaggggtccctgaat	UPL_#33	80
