# Supplementary material for: A Semi-automated Organoid Screening Method Demonstrates Epigenetic Control of Intestinal Epithelial Differentiation
Source: Front Cell Dev Biol. 2021 Jan 21;8:618552. doi: 10.3389/fcell.2020.618552 (PMC7872100; doi:10.3389/fcell.2020.618552)
Supplement: Supplementary Table 3 — Materials, reagents, and software used. [file Data_Sheet_4.pdf]

Table 3:
Materials, Reagents, Software

Item	Supplier	Specification	Publication	Comment
**Mice**				
C57BL/6JRj wild type	Janvier Labs			
C57BL/6 wild type				
B6D2 (C57Bl/6 x DBA/2) *Villin* -Cre			elMarijou2004	kind gift from Sylvie Robine, Institut Curie-CNRS, Paris, France
C57BL/6J *Lsd1* $^{f/f}$			Kerenyi2013	kind gift from Stuart Orkin, Harvard Medical School , Boston , USA
B6.129P2 *Apc* ^{tm1Rsmi}/RfoJ	Jackson Laboratories	stock no: 029275		
Lgr5 -EGFP-IRES-CreERT2	Jackson Laboratories	stock no: 008875		
Hpgds -tdTomato			Bornstein2018	
Neurog3 -RFP			Kim2015	kind gift from Anne Grapin-Botton, DanStem, University of Copenhagen, Copenhagen, Denmark
**Cell lines**				
cell line producing Noggin				kind gift from Hans Clevers, Hubrecht Institute, Utrecht, The Netherlands
cell line producing R-Spondin				kind gift from Calvin Kuo, Stanford University School of Medicine, Stanford, USA
**Plates**				
24-well plate	Corning	#3523		
96-well glass-bottom plate	Cellvis	#P-96-1N		
384-well optical plates	Applied Biosystems	#4309849		
8-well microscopy slide	Ibidi	#80821		
**Reagents**				
PBS	Sigma-Aldrich	#806552		
EDTA	Thermo Fisher Scientific	#15575020		
TrypLE Express	Thermo Fisher Scientific	#12605010		
Tamoxifen	Sigma-Aldrich	#T5648		
Corn oil	Sigma-Aldrich	#C8267		
FCS	Gibco	#10270106		
Saponin	Sigma-Aldrich	#8047-15-2		
Triton X-100	Sigma-Aldrich	#T8787		
PFA	Alfa Aesar	#43368		
**Organoid culture**				
Matrigel	Corning	#734-1101		
DMEM F12	Gibco	#31330-038		
Penicillin-Streptomycin	Sigma-Aldrich	#P0781		
HEPES	Gibco	#35050061		
Glutamax	Gibco	#35050061		
B-27 supplement	Gibco	#35050061		
N2 supplement	Gibco	#17502001		
N-Acetylcysteine	Sigma-Aldrich	#A-7250		
EGF Recombinant Mouse Protein	Thermo Fisher Scientific	#PMG8041		
CHIR99021	Sigma-Aldrich	#SML1046		
Valproic acid (VPA)	Cayman Chemicals	#13033		
DAPT	Cayman Chemicals	#13197		
IWP-2	Cayman Chemicals	#13951		
**RNA isolation, qRT-PCR**				
RNA-solv Reagent	Omega Bio-Tek	#R6830-02		
Direct-zol MiniPrep kit	Zymo Research	#R2051		
Direct-zol-96	Zymo Research	#R2055		
High-Capacity RNA-to-cDNA Kit	Applied Biosystems	#4388950		
2x Perfecta ROX,UNG Fast Mix	Quanta Biosciences	#733-1398		
Primers	Sigma-Aldrich			see Supplementary Table 2
Universal ProbeLibrary probes	Roche			see Supplementary Table 2
**Antibodies**				
**IF & IHC staining reagents**				
BD CompBead Anti-Mouse Ig	Becton Dickinson	#552843		
BD CompBead Anti-Rat and Anti-Hamster Ig	Becton Dickinson	#552845		
DAPI	Thermo Fisher Scientific	#62248		
Hoechst 33342	Thermo Fisher Scientific	#62249		
Zombie Aqua	Biolegend	#423102		
TruStain FcX	Biolegend	#101320		
CD24-PerCp-Cy5.5, clone M1/69	Biolegend	#101824		
CD24-AF647, clone M1/69	Biolegend	#101818		
CD44-AF647, clone IM7	Biolegend	#103018		
CD44-BV785, clone IM7	Biolegend	#103041		
CD117-PE-Cy7, clone ACK2	Biolegend	#135112		
CD326-BV421, clone G8.8	Biolegend	#118225		
CD326-BV605, clone G8.8	Biolegend	#118227		
UEA1-FITC, Ulex Europaeus Agglutinin I	Invitrogen	#L32476		
UEA1-Rhodamine, Ulex Europaeus Agglutinin I	Vector Laboratories	#RL-1062-2		
Goat Anti-Rabbit IgG-BV421	Invitrogen	#A-31556		
Goat Anti-Rabbit IgG-AF488	Invitrogen	#A-11034		
anti-DCLK1	Abcam	#ab31704		
anti-MUC2	Santa Cruz	#sc-15334		
anti-KI67	Invitrogen	#MA5-14520		
Fluoromount G	Invitrogen	#00-4958-02		
EnVision-HRP	Dako	#K4063 & #K4061		
DAB	Dako	#K5007		
**mRNA sequencing**				
Quick-RNA MicroPrep kit	Zymo Research	#R1050		
Illumina TruSeq Stranded Total RNA	Illumina			I-CBP112 study, carried out at Genomics Core Facility, NTNU
Illumina NS500 MO flow-cell	Illumina			I-CBP112 study, carried out at Genomics Core Facility, NTNU
NEB Next Ultra RNA Library Prep Kit	NEB			MS023 study, carried out at Novogene (UK) Co
**Instruments**				
EVOS FL Auto2 microscope	Thermo Fisher Scientific			
Eclipse Ci-L microscope	Nikon			
LSM880 confocal microscope	Zeiss			
Axio Imager Z1 confocal microscope	Zeiss			
NanoDrop-1000	NanoDrop			
QuantStudio 5 Real-Time PCR system	Thermo Fisher Scientific			
BD LSRII flow cytometer	Becton Dickinson			
BD FACSAria III flow cytometer	Becton Dickinson			
MACSQuant X flow cytometer	Miltenyi Biotec			
Viaflo 96-channel pipette	Integra Biosciences			
Qubit Fluorometric Quantitation system	Life Technologies			
2100 Bioanalyzer	Agilent			
Ilumina NextSeq 500	Ilumina			
Illumina NovaSeq 6000	Illumina			

**Software**			
GNU R		v3.6.3	
Bioconductor		v3.10	
tidyverse		v1.3.0	Wickham2019
ggpubr		v0.3.0	
ggimage		v0.2.8	
magick		v2.3	
pheatmap		v1.0.12	
ggtree		v2.0.4	Yu2018
flowCore		v1.53.17	
CytoML/flowWorkspace		v1.12.1	
ggcyto		v1.14.1	Van2018
flowViz		1.50.0	
biomaRt		v2.42.1	Durinck2005
ClusterProfiler		v3.14.3	Yu2012
ImageJ/Fiji		v1.52n	Schindelin2012
Ilastik		v1.3.2	Berg2019
FlowJo software	FlowJo, LLC	v10.6.2	
QuantStudio Design & Analysis Software	Thermo Fisher Scientific	v1.5.1	
FastQC		v0.11.8	
MultiQC		v1.7	Ewels2016
featureCounts		v1.6.4	Liao2014
GNU R		v3.6.1	
STAR		v2.7.3a	Dobin2013
DESeq2		v1.26.0	Love2014
**Datasets**			
Ensembl		GRCh38.p13	
GENCODE annotation		M18	Frankish2019
STRING-DB		v11	Szklarczyk2019
TRRUST		v2	Han2018
IEC lineage signatures		GSE92332	Haber2017
LGR5+ stem cells		GSE33949	Munoz2012
GO:BP		GO Consortium	TheGeneOntologyConsortium2019
tpm BRDs, PRMTs		E-MTAB-9077	Zwiggelaar2020
tpm BRDs, PRMTs		E-MTAB-78	Zwiggelaar2020
scRNA tpm Prmt1		GSE92332	Haber2017
